# Replications of Two Closely Related Groups of Jumbo Phages Show Different Level of Dependence on Host-encoded RNA Polymerase

**DOI:** 10.3389/fmicb.2017.01010

**Published:** 2017-06-13

**Authors:** Takeru Matsui, Genki Yoshikawa, Tomoko Mihara, Orawan Chatchawankanphanich, Takeru Kawasaki, Miyako Nakano, Makoto Fujie, Hiroyuki Ogata, Takashi Yamada

**Affiliations:** ^1^Department of Molecular Biotechnology, Graduate School of Advanced Sciences of Matter, Hiroshima UniversityHigashi-Hiroshima, Japan; ^2^Bioinformatics Center, Institute for Chemical Research, Kyoto UniversityKyoto, Japan; ^3^Plant Research Laboratory, National Center for Genetic Engineering and Biotechnology, NSTDAPathum Thani, Thailand; ^4^Center for Agricultural Biotechnology, Kasetsart UniversityNakhon Pathom, Thailand

**Keywords:** jumbo phages, ΦKZ-like phages, *Ralstonia solanacearum*, genomic analysis, virion-associated-RNA polymerase

## Abstract

*Ralstonia solanacearum* phages ΦRP12 and ΦRP31 are jumbo phages isolated in Thailand. Here we show that they exhibit similar virion morphology, genome organization and host range. Genome comparisons as well as phylogenetic and proteomic tree analyses support that they belong to the group of ΦKZ-related phages, with their closest relatives being *R. solanacearum* phages ΦRSL2 and ΦRSF1. Compared with ΦRSL2 and ΦRSF1, ΦRP12 and ΦRP31 possess larger genomes (ca. 280 kbp, 25% larger). The replication of ΦRP12 and ΦRP31 was not affected by rifampicin treatment (20 μg/ml), suggesting that phage-encoded RNAPs function to start and complete the infection cycle of these phages without the need of host-encoded RNAPs. In contrast, ΦRSL2 and ΦRSF1, encoding the same set of RNAPs, did not produce progeny phages in the presence of rifampicin (5 μg/ml). This observation opens the possibility that some ΦRP12/ΦRP31 factors that are absent in ΦRSL2 and ΦRSF1 are involved in their host-independent transcription.

## Introduction

“Jumbo phages” are bacteriophages, classified in the Myoviridae family, with a large genome over 200 kbp (Hendrix, 2009; Yuan and Gao, 2017). Currently isolated examples include *Pseudomonas aeruginosa* phage ΦKZ (280 kbp, Mesyanzhinov et al., 2002) and EL (211 kbp, Hertveldt et al., 2005), *Pseudomonas chlororaphis* phage 201Φ2-1 (317 kbp, Thomas et al., 2008), *Pseudomonas fluorescens* phage OBP (284 kbp, Cornelissen et al., 2012), *Stenotrophomonas maltophilia* phage ΦSMA5 (250 kbp, Chang et al., 2005), *Vibrio parahaemolyticus* phage KVP40 (386 kbp, Miller et al., 2003), *Yersinia enterocolitica* phage R1-37 (270 kbp, Kiljunen et al., 2005), *Klebsiella* phage vB_KleM-RaK2 (346 kbp, Simoliunas et al., 2013), and *Bacillus* phage AR9 (251 kbp, Lavysh et al., 2016). Jumbo phages have also been isolated from plant-associated bacteria. Such phages include *Sinorhizobium meliloti* phage N3 (207 kbp, Martin and Long, 1984), *Erwinia amylovora* phage PhiEaH1 (218 kbp, Meczker et al., 2014), and vB_Eam_Ea35-70 (271 kbp, Yagubi et al., 2014), and *Ralstonia solanacearum* phage ΦRSL1 (240 kbp, Yamada et al., 2010), ΦRSL2 and ΦRSF1 (220 kbp, Bhunchoth et al., 2016). *Bacillus megaterium* phage G possesses so far the largest sequenced genome (498 kbp; accession no. JN638751; Sun and Serwer, 1997). Some of the sequenced jumbo phages are known to encode many proteins with considerable similarity to ΦKZ proteins, and are called ΦKZ-related phages (Cornelissen et al., 2012; Jang et al., 2013). One of the notable features of ΦKZ-related phages is the independence of their replication from the host transcriptional machinery. This property is attributed to two sets of phage-encoded multisubunit RNA polymerase (RNAP) subunits (β- and β′- subunits) (Ceyssens et al., 2014; Yukunina et al., 2015; Lavysh et al., 2016). It has been suggested that transcription of the ΦKZ genome proceeds by the consecutive action of these two sets of RNAPs, one of which (virion-associated-RNAP) is packed within the virion, introduced into the host cytoplasm with the genomic DNA upon infection, and employed for transcription of early genes, and the other (early-expressed-RNAP) is the product of early genes and is employed for transcription of middle and late genes (Ceyssens et al., 2014). ΦKZ replication in *P. aeruginosa* was demonstrated to be resistant to rifampicin treatment (400 μg/ml). A rifampicin resistant multisubunit RNAP was also reported in *Bacillus subtilis* infected with phage PBS2 (Clark et al., 1974). PBS2 is a clear plaque derivative of PBS1 (Takahashi, 1963) and closely related to AR9 (Rima and van Kleeff, 1971). AR9, belonging to ΦKZ-related phages, was recently shown to encode two sets of β- and β′-subunits of RNAP and its infection was shown to be resistant to rifampicin (Lavysh et al., 2016). Recently, two *R. solanacearum* phages, ΦRSL2 and ΦRSF1 were characterized as ΦKZ-related viruses (Bhunchoth et al., 2016). All β- and β′-subunits of virion-associated-RNAP were detected in ΦRSF1 particles except for one β′-subunit undetected in ΦRSL2 particles. In contrast to ΦKZ and AR9, however, the replication of both ΦRSL2 and ΦRSF1 were inhibited by rifampicin (Bhunchoth et al., 2016). These results suggest functional variations of the phage-encoded multisubunit RNAPs among different KZ-related phages. In this work, we show that two newly isolated jumbo phages infecting *R. solanacearum* are closely related to ΦRSL2/ΦRSF1 but their infection is resistant to rifampicin treatment.

## Materials and methods

### Bacterial strains, bacteriophages, and culture conditions

*R. solanacearum* strains used in this study, their plant hosts and taxonomic characteristics are shown in Supplementary Table S1. Bacteria were cultured in CPG medium containing 0.1% (w/v) casamino acids, 1.0% (w/v) peptone, and 0.5% (w/v) glucose (Horita and Tsuchiya, 2002) at 28°C with shaking at 200–300 rpm. Bacteriophages ΦRP12 and ΦRP31 were isolated from tomato fields in Chiang Mai, Thailand as described previously (Bhunchoth et al., 2015). Each phage was routinely propagated using *R. solanacearum* strain MAFF 730138 as the host. When the cultures reached an OD_600_ of 0.5, bacteriophages were added at a multiplicity of infection (MOI) of 0.01–0.1. After culturing for a further 12–24 h, the cells were removed by centrifugation at 8,000 × *g* for 15 min at 4°C. The supernatant was membrane-filtered (0.45-μm pore; Steradisc, Kurabo Co. Ltd., Osaka, Japan), and the pellet was dissolved in SM buffer (50 mM Tris-HCl at pH 7.5, 100 mM NaCl, 10 mM MgSO_4_, and 0.01% gelatin). For further purification, the phage suspension was layered on a linear 20–60 % sucrose gradient and centrifuged at 40,000 × *g* for 1 h. The purified phages were stored at 4°C. Phage titers were determined by a plaque-forming assay, with *R. solanacearum* MAFF 730138 as the host, on CPG plates containing 1.5% agar overlaid with 0.45% CPG soft agar. For electron microscopic observation, the phage particles were stained with Na-phosphotungstate and analyzed using a JEOL JEM-1400 electron microscope (JEOL Ltd., Tokyo, Japan) according to Dykstra (1993). λ phage particles were used as an internal standard marker for size determination.

### Single-step growth experiments and treatment with rifampicin

Single-step growth experiments were performed as previously described (Yamada et al., 2010), with some modifications as follows: Bacterial cells (strain MAFF 730138 as the host) at 0.1 U of OD_600_ were harvested by centrifugation at 8,000 × *g* for 15 min at 4°C and resuspended in 10 ml fresh CPG medium (approximately 1 × 10^8^ colony-forming units (CFU)/ml). The cells were added with phage at a MOI of 0.1 and allowed to adsorb for 10 min at 28°C. After centrifugation at 8,000 × *g* for 15 min at 28°C, samples were resuspended in the initial volume of CPG, and serial dilutions were made in a final volume of 10 ml. During incubated at 28°C, samples were removed at 30-min intervals up to 5 h and the titers were determined using double-layer plaque assay.

For rifampicin treatment, freshly growing 5 ml cultures of MAFF 730138 (at OD_600_ = 0.1) were added with rifampicin (at various concentrations). Twenty min after the addition, the cultures were infected with phages at MOI = 1.0 and incubated for 20 h at 28°C with shaking at 200–300 rpm. Phage titers were assayed as described above. For control phages, a myovirus ΦRSL1 (Yamada et al., 2010) and a podovirus ΦRSB1 (Kawasaki et al., 2009) were used.

### Isolation and sequencing of genomic DNA from phage particles

DNA purification, digestion with restriction enzymes, and sequencing were performed following Sambrook and Russell (2001). To determine whole genome size by pulsed-field gel electrophoresis (PFGE), the purified phage particles were embedded in 0.5% low-melting-point agarose (InCert agarose, FMC Corp., Philadelphia, PA, USA). Following treatment with proteinase K (1 mg/ml; Merck Ltd., Tokyo, Japan) and 1% (w/v) sarkosyl, the nucleic acids were subjected to PFGE using a CHEF Mapper electrophoresis apparatus (Bio-Rad, Hercules, CA, USA) as described by Higashiyama and Yamada (1991). Genomic DNA was extracted from the purified phage particles by phenol extraction. Shotgun sequencing of phage DNA was performed at Hokkaido System Science Co., Ltd. (Sapporo, Japan) using a Roche GS Junior System. Draft sequences were assembled using GS De Novo Assembler, version 2.6. The sequence depth was 532 and 983 times the final contig sizes of ΦRP12 (279,845 bp) and ΦRP31 (276,958 bp), respectively.

### Bioinformatics

ORFs were identified using GeneMarkS version 4.32 using ATG, GTG, and TTG as possible start codons (Besemer et al., 2001). Homology searches were performed using BLASTP/RPS-BLAST (Altschul et al., 1997) against UniProt sequence database (UniProt Consortium, 2015), NCBI/Cdd sequence domain database version 3.15 (Wheeler et al., 2007), and NCBI RefSeq complete viral genome database (Release 76) by applying an *E*-value cutoff of 1e-5. PSI-BLAST searches were also performed using 201Φ2-1 amino acid sequences as queries and NCBI RefSeq complete viral genome database as a target database with five iterations (with options -inclusion_ethresh 1e-5 and -evalue 1e-5). tRNAScan-SE 1.3.1 (option: -B for bacterial tRNAs) was used to identify tRNA genes (Lowe and Eddy, 1997). Circular genome maps were generated using CGView (Stothard and Wishart, 2005) and dot-plots by an in-house script. Sequences were aligned using MAFFT v7.220 (Katoh and Standley, 2016) with default parameters. Evolutionary model for phylogenetic reconstruction was selected using ProteinModelSelection.pl of RaxML. Selected models were LGF for both tail sheath proteins and terminases. Tree reconstruction was performed using RaxML v8.2.4 (Stamatakis, 2014) with the selected model and PROTGAMMA parameter with 100 bootstrap replicates. Proteomic tree reconstruction was performed as previously described (Bhunchoth et al., 2016).

### Identification of virion proteins by liquid chromatography-tandem mass spectrometry

Purified phage particles were subjected to SDS-polyacrylamide gel electrophoresis (SDS-PAGE) (10–12% polyacrylamide) according to Laemmli (1970). After staining with Coomassie Brilliant Blue, protein bands were excised from the gel, and digested with trypsin. Tryptic peptides trapped with a short ODS column (PepMap 100; 5 μm C18, 5 mm × 300 μm ID, Thermo Fisher Scientific Inc., Waltham, MA, USA) were then separated with another ODS column (Nano HPLC Capillary Column; 3 μm C18, 120 mm × 75 μm ID, Nikkyo Technos, Tokyo, Japan) using nano-liquid chromatography (Ultimate 3000 RSLC nano system, Thermo Fisher Scientific Inc.) according to Bhunchoth et al. (2016). The eluate was then continuously introduced into a nanoESI source and analyzed by mass spectrometry (MS) and MS/MS (LTQ Orbitrap XL, Thermo Fisher Scientific Inc.). The MS and MS/MS spectra were generated in the positive ion mode using Orbitrap (mass range: *m/z* 300–1,500) and Iontrap (data-dependent scan of the top five peaks using CID), respectively. The capillary source voltage was set at 1.5 kV, and the transfer capillary temperature was maintained at 200°C. The assignment of the MS/MS data to tryptic peptides encoded by phage ORFs was completed as previously described (Ahmad et al., 2014) using the Xcalibur program, version 2.0 (Thermo Fisher Scientific Inc.). All MS/MS data were searched using Mascot (Matrix Sciences) against the GeneBank non-redundant protein database and against an in-house database of all possible ΦRP12/ΦRP31 gene products using Proteome Discoverer software (ver. 1.4, Thermo Fisher Scientific Inc.). Doubly, triply and quadruply charged peptide ions were subjected to the database search with a parent and peptide ion mass tolerance of ±10 ppm and ±0.8 Da, respectively. Cysteine carbamidomethylation, methionine oxidation and deamidation of asparagine and glutamine were possible static and chemical modifications. The significance threshold on Proteome Discoverer for Mascot search was set at *P* < 0.05 and one and two missed trypsin cleavage was allowed. Proteomics raw data and search files for protein identification of ΦRP31 have been deposited to the ProteomeXchange Consortium (announced ID: PXD006355) via the jPOST partner repository (announced ID: JPST000264).

## Results

### Isolation and initial characterization of ΦRP12 and ΦRP31

ΦRP12 and ΦRP31 were isolated from soil samples collected from tomato fields in Chiang Mai, Thailand. They formed very small clear plaques (<0.1 mm) with host strains on 0.45% top agar plates, but formed larger plaques (1–2 mm) when the top agar concentration was decreased to 0.3%. Both phages showed the same host range, infecting 14 of 21 tested *R. solanacearum* strains isolated in Japan (Supplementary Table S1). The jumbo phage nature of ΦRP12 and ΦRP31 was recognized by their large genome size and morphology. In pulsed-field gel electrophoresis analyses, the genomic DNA of these phages gave a single band of approximately 270–280 kbp, being considerably larger than those of previously isolated *Ralstonia* jumbo phages such as ΦRSL1 (240 kbp) and ΦRSL2 (220 kbp) (Supplementary Figure S1). Morphological features of ΦRP12 and ΦRP31 particles revealed by electron microscopy were indistinguishable with each other and were characteristic to myoviruses, with an icosahedral head (diameter: 120 ± 5 nm, *n* = 10) and a long contractile tail (length: 180 ± 10 nm, *n* = 10; width: 25 ± 2 nm, *n* = 10, respectively) (Supplementary Figure S2). The ΦRP12 and ΦRP31 particles were very similar to those of ΦRSL2 and ΦRSF1 (Bhunchoth et al., 2016).

### General genomic features of ΦRP12 and ΦRP31

**Figure 1:**
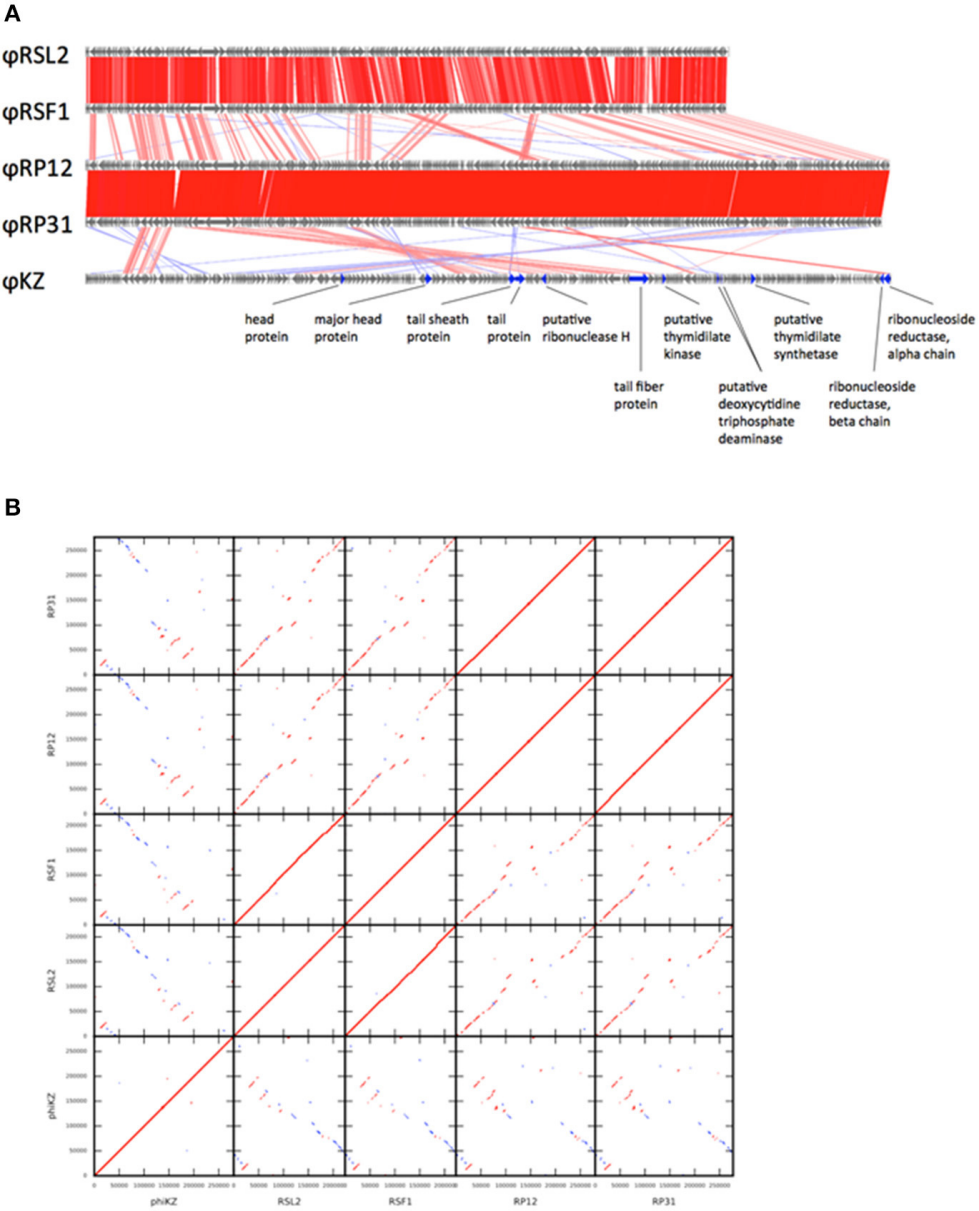
Genome comparison among five ΦKZ-related phage genomes. **(A)** Linear genome alignment of five ΦKZ-related phages. Red and blue lines between genomes represent sequence similarities (≥50% identity) detected by TBLASTX in the same and reverse orientations, respectively. **(B)** Dot-plot comparison among five ΦKZ-related phages. Red and blue lines represent sequence similarities detected by TBLASTX in the same and reverse orientations, respectively.

Phage genomic sequences were assembled into a circular contig of 279,845 bp for ΦRP12 (accession no. AP017924) and 276,958 bp for ΦRP31 (accession no. AP017925), respectively. G+C contents of the ΦRP12 and ΦRP31 genomes were 53.40 and 53.35 %, respectively, which were significantly lower than that of the host genome (e.g., 66.97% for *R. solanacearum* strain GMI1000; accession no. NC_003295). The genomes of the two phages resembled each other and exhibited nearly perfect co-linearity (Figures 1A,B). In total, 289 and 287 open reading frames (ORFs) were predicted in the genomes of ΦRP12 and ΦRP31, respectively (Supplementary Table S2). The average sequence identity between the ΦRP12 and ΦRP31 ORFs was 99.13% at the amino acid sequence level and 98.80% at the nucleotide sequence level. A tRNA gene for Asn (GTT) was detected in both genomes. In accordance with the high level of ORF sequence similarity (Figures 1A,B), the gene order was highly conserved between ΦRP12 and ΦRP31, although several genes were specific to one of the genomes (Supplementary Table S2). Fifteen percent (43/289) of ΦRP12 ORFs and 15.7% (45/287) of ΦRP31 ORFs were located in a clockwise direction, with the remaining ORFs encoded in a counterclockwise direction in the circular maps shown in Supplementary Figures S3A,B. Based on systematic database searches, 77 and 74 ORFs of ΦRP12 and ΦRP31, respectively, were functionally annotated (Supplementary Tables S2). Many ΦRP12 and ΦRP31 ORFs showed significantly similarities to ΦKZ-related phage ORFs. For instance, 53 ORFs of ΦRP12 showed their best hit to ORFs in ΦKZ, ΦRSL2, or ΦRSF1 (average amino acid sequence identity, 34.5%) (Bhunchoth et al., 2016).

### Evolutionary relationships between ΦRP12/ΦRP31 and ΦKZ-related phages

PSI-BLAST searches identified 95 ORFs showing significant sequence similarities to 201Φ2-1 ORFs for each of ΦRP12 and ΦRP31. These numbers are greater than those reported for two ΦKZ-related phages, OBP (67 ORFs) and EL (69 ORFs) (Bhunchoth et al., 2016). The genomes of ΦRP12 and ΦRP31 showed conserved co-linear segments with the genomes of ΦRSL2 and ΦRSF1 along their entire lengths, except their central regions (Figure 1A: genome alignment, Figure 1B: dot-plots). In contrast, ΦRP12/ΦRP31 showed much more fragmented but still recognizable co-linearity when compared with ΦKZ. These genomic similarities suggest evolutionarily close relationships of ΦRP12/ΦRP31 with ΦKZ-related phages, especially ΦRSL2/ΦRSF1.

**Figure 2:**
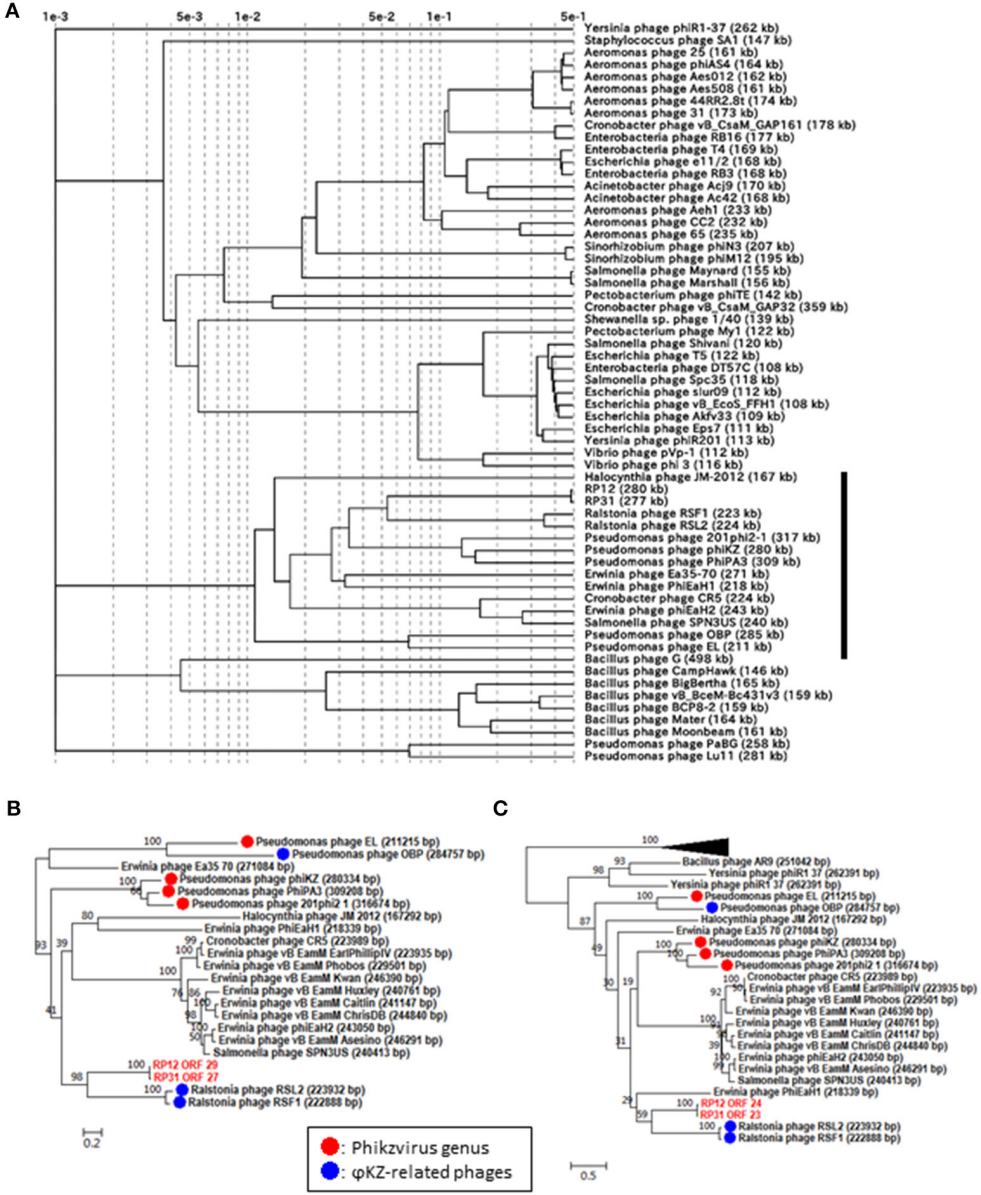
Proteomic and phylogenetic relationships between ΦRP12/ΦRP31 and other phages. **(A)** A proteomic tree produced by the BIONJ program (Gascuel, 1997) based on TBLASTX genomic sequence comparisons of 61 phage genomes. Branch lengths from the root were scaled logarithmically. In this logarithmic representation, nodes that were at distances smaller than 0.001 from the root were agglomerated into the root point. **(B,C)** Maximum likelihood phylogenetic trees of the tail sheath and terminase large subunit proteins, respectively. Statistical support at node is given as bootstrap values. Number at scale bar indicates the number of substitutions per site.

In order to corroborate the hypothesized evolutionary link between ΦRP12/ΦRP31 and ΦKZ-related phages, we carried out a proteomic and phylogenetic tree reconstructions. A phage proteomic tree based on previously reported method (Bhunchoth et al., 2016) revealed a relatively compact clade composed of 15 phages (Figure 2A). These phages are ΦRP12/ΦRP31, ΦKZ-related phages, and other phages previously reported as being related to ΦKZ-related phages (Cornelissen et al., 2012; Jang et al., 2013; Bhunchoth et al., 2016). Among the 15 phages, ΦRP12/ΦRP31 formed a subclade with ΦRSL2/ΦRSF1, which were together closely related with phages of the *Phikzvirus* genus (e.g., ΦKZ, 201Φ2-1). We also constructed maximum likelihood phylogenetic trees for phage genes (tail sheath proteins, Figure 2B and terminases, Figure 2C). Again, compared to *Pseudomonas* phages OBP and EL, ΦRP12 and ΦRP31 forming a clade with ΦRSL2 and ΦRSF1 were closer to phages of the *Phikzvirus* genus. Based on these genomic similarities, proteomic tree, and gene phylogenies, we propose that ΦRP12 and ΦRP31 represent new members of the ΦKZ-related phage group.

Phages with a genome larger than 200 kb were scattered across the proteomic tree, suggesting multiple evolutionary origins of diverse jumbo phages as previously proposed (Bhunchoth et al., 2016, Figure 2A). Of the 15 phages forming the above mentioned clade containing ΦKZ-related phages, 14 phages have genomes greater than 200 kb. This suggests that the large genome size of this group has been stable during the course of evolution in spite of genomic rearrangements that altered their gene order and contents.

### ΦRP12 and ΦRP31 gene annotations

Notable genes found in the genomes of ΦRP12 and ΦRP31 are described as follows.

**Figure 3:**
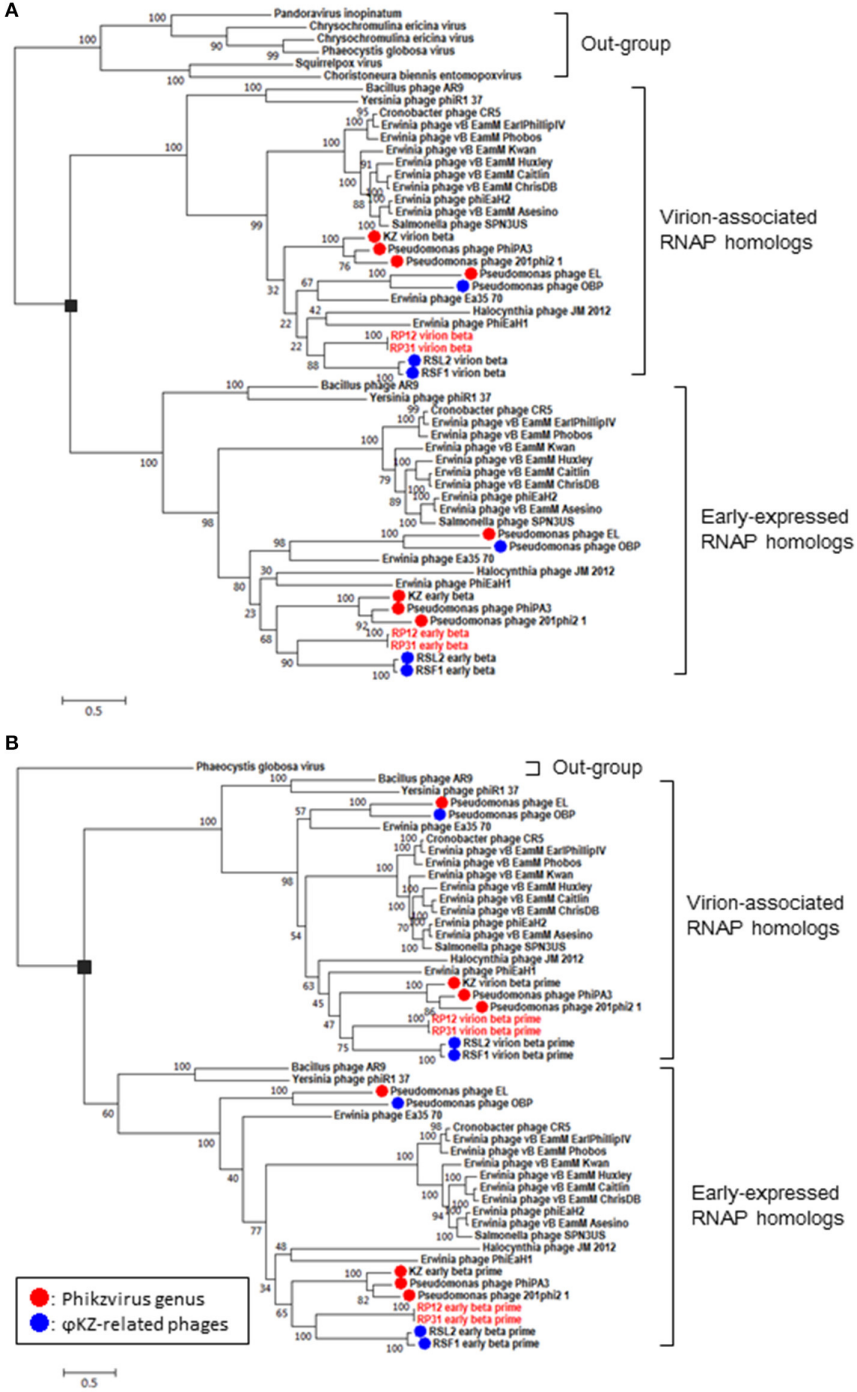
Phylogenetic relationships of virion-associated and early-expressed RNAP homologs. Maximum likelihood phylogenetic trees of RNA polymerase β subunits **(A)** and β′ subunits **(B)**. ORFs corresponding to each subunit were concatenated before building sequence alignments. Black rectangles correspond to proposed gene duplications. Bootstrap values are given along the branches. Number at scale bar indicates the number of substitutions per site.

**Figure 4:**
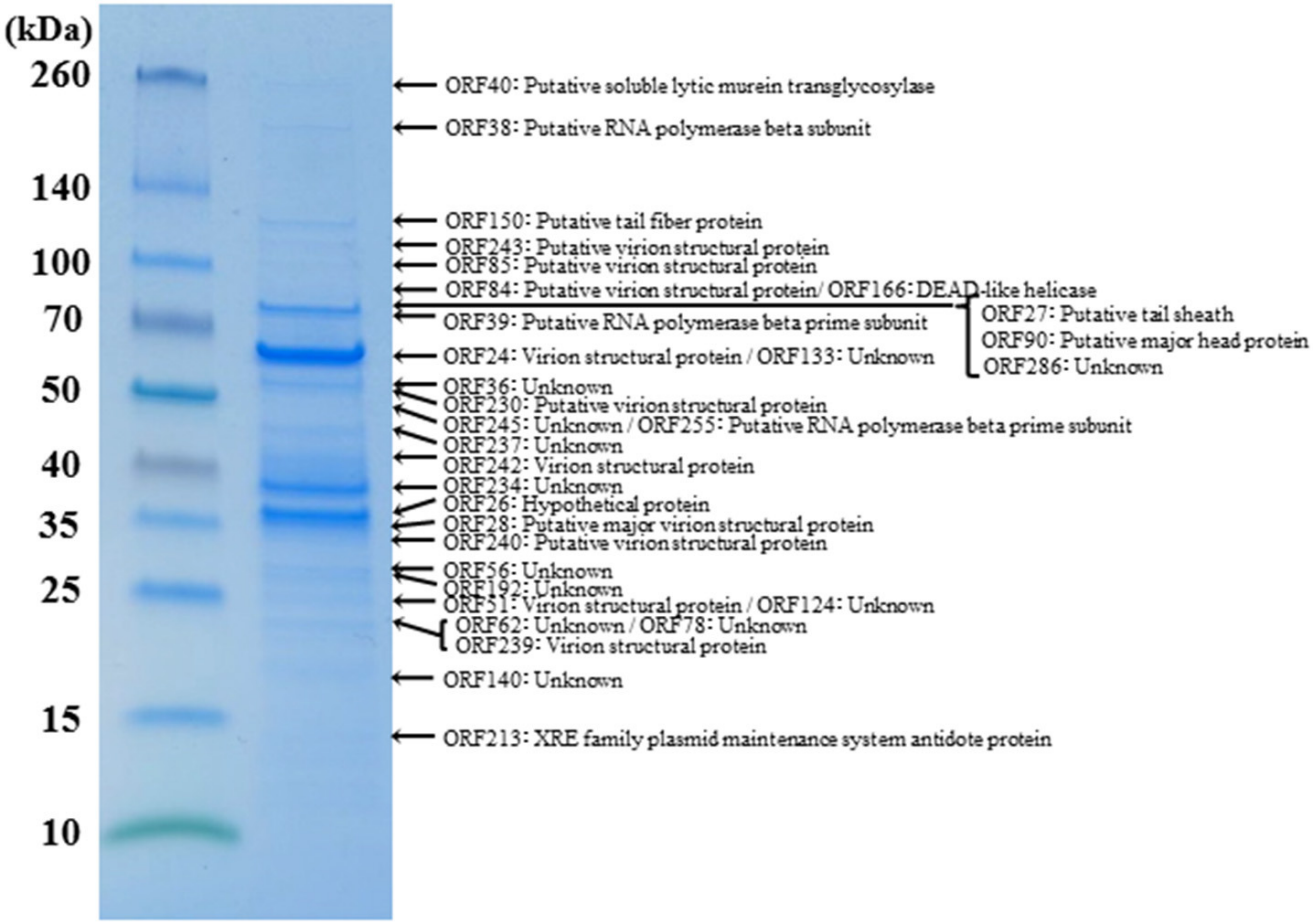
Proteomic analysis of virion proteins of ΦRP31. Proteins from purified ΦRP31 particles were separated by SDS-PAGE and stained with Coomassie blue. The protein bands excised from the SDS-PAGE gel were subjected to trypsin digestion and analysis by liquid chromatography-tandem mass spectrometry (LC-MS/MS, LTQ Orbitrap XL). Tandem mass spectrometry data were assigned to tryptic peptides encoded by phage open reading frames using an established procedure (Ahmad et al., 2014). Asterisks indicate the fragmented β and β′ subunits of virion-associated-RNAP. VSP: virion structural protein.

1. **RNA polymerase β- and β**′**-subunits**. During the infection cycle of ΦKZ, two distinct multisubunit RNAPs were proposed to function: a virion-packed-RNAP, which is middle expressed and responsible for early gene expression in the absence of host RNAP activity, and another early-expressed RNAP, which functions for middle and late phases of gene expression (Ceyssens et al., 2014). All genes corresponding to the ΦKZ-RNAP β- and β′-subunits (virion-associated-RNAP, Gp80, Gp149, Gp178, and Gp180 as well as early-expressed-RNAP, Gp55, Gp71-Gp73, Gp74, and Gp123) were identified in both ΦRP12 and ΦRP31 genomes. The possible orthologous genes among these phages, ΦRSL2 and ΦRSF1 are shown in Table 1. All proteins corresponding to ΦKZ virion-associated-RNAP subunits were detected in the ΦRP31 virion (see below). Our phylogenetic analyses indicate that virion-associated-RNAP and early-expressed-RNAP genes form distinct clades and that, inside each of the clades, phylogenetic relationships between different phages were similar. This suggests that virion-associated-RNAP homologs and early-expressed-RNAP homologs arose as a result of gene duplications that occurred in an ancestral virus that diverged to ΦKZ-related phages and other phages with relatively large genomes including *Bacillus* phage AR9 (Figure 3).
2. **Proteins involved in DNA replication, recombination, and repair**. ΦRP12 and ΦRP31 predicted proteins involved in DNA replication included a T4-like DNA polymerase (ΦRP12-ORF3 and -ORF251 and ΦRP31-ORF2 and -ORF248), an RNase H (ΦRP12-ORF62 and ΦRP31-ORF58), UvsX (ΦRP12-ORF59 and ΦRP31-ORF55), a SbcC-ATPase (ΦRP12-ORF69 and ΦRP31-ORF65), SbcD (ΦRP12-ORF282 and ΦRP31-ORF279), a DNA ligase (ΦRP12-ORF161 and ΦRP31-ORF158), a crossover junction endonuclease (ΦRP12-ORF86 and ΦRP31-ORF82), a DnaB helicase (ΦRP12-ORF97 and ΦRP31-ORF92), a DEAD-like helicase (ΦRP12-ORF169 and ΦRP31-ORF166), and a RAD2/SF2 helicase (ΦRP12-ORF267 and ΦRP31-ORF264). ΦRP12-ORF54 was similar to GIY-YIG type nucleases, which are often involved in transfer of mobile genetic elements and/or DNA repair and recombination.
3. **Nucleotide metabolism and DNA modification enzymes**. ΦRP12 and ΦRP31 encoded at least eight predicted enzymes for nucleotide metabolisms, including a dCTP deaminase (ΦRP12-ORF134 and ΦRP31-ORF131), a ribonucleotide reductase α subunit (ΦRP12-ORF156 and ΦRP31-ORF153) and a β subunit (ΦRP12-ORF155 and ΦRP31-ORF152), a dihydrofolate reductase (ΦRP12-ORF185 and ΦRP31-ORF182), a nicotinate phosphoribosyltransferase (ΦRP12-ORF211 and ΦRP31-ORF208), a ribose-phosphate pyrophosphokinase (ΦRP12-ORF212 and ΦRP31-ORF209), a thymidylate synthase (ΦRP12-ORF215 and ΦRP31-ORF212), and a thymidylate kinase (ΦRP12-ORF221 and ΦRP31-ORF218). In either genome, we identified no genes for enzymes involved in DNA modification, such as adenine and cytosine methylation or cytosine hydroxymethylation.
4. **Lysis and host-phage interaction**. ΦRP12-ORF43 and ΦRP31-ORF40 were similar to soluble lytic murein transglycosylases (chitinase-like glycosylases or glycoside hydrolase). They were homologous to the putative cell-puncturing protein Gp181 (2,237 amino acids) of ΦKZ (Fokine et al., 2007). Proteins encoded by ΦRP12-ORF166 and ΦRP31-ORF163 showed similarities to the lytic transglycosylase-like proteins. Soluble transglycosylases of this type degrade murein *via* cleavage of the β-1,4-glycosidic bond between N-acetylmuramic acid and N-acetylglucosamine, concomitantly forming a 1,6-anhydro bond in the muramic acid residue. ΦRP12-ORF265 and ΦRP31-ORF262 encode peptideglycan binding motifs and may be involved in host-phage interactions. AR9 was previously found to encode a putative holin gene (*g082*) (Lavysh et al., 2016) but no homologs were found in ΦRP12/ΦRP31.
5. **Virion structural proteins**. A comparative analysis of the ΦRP12 and ΦRP31 genome sequences enabled the annotation of 29 and 28 structure-related genes, respectively (Supplementary Table S2). The structural genes included those for major capsid proteins (ΦRP12-ORF30 and –ORF94 and ΦRP31-ORF28 and –ORF90), cell puncturing device (ΦRP12-ORF43 and ΦRP31-ORF40), tail fiber (ΦRP12-ORF153 and ΦRP31-ORF150), tail sheath (ΦRP12-ORF29 and ΦRP31-ORF27), and other possible structural phage proteins. Reversed-phase nano-liquid chromatography directly coupled with liquid chromatography-tandem mass (LC-MS/MS) spectrometry analysis of the proteins of ΦRP31 virion separated by SDS-PAGE resulted in the identification of 32 ΦRP31 virion proteins, all of which had orthologs in ΦRP12 (Figure 4 and Supplementary Table S3). These included 61% (17/28) of the ΦRP31 ORFs that were predicted to encode virion-associated proteins described above (Supplementary Table S2B) and additional proteins showing marginal homology to some enzymes and other unknown functions. All β-subunits (ORF38 and ORF51) and β′-subunits (ORF39 and ORF255) of virion-associated-RNAP were detected in ΦRP31 (Figure 4 and Supplementary Table S3). LC-MS/MS analysis was not performed for ΦRP12.
6. **Other genes**. Several ORFs encoding proteins homologous to known functional proteins were detected in ΦRP12 and ΦRP31, including a MutT/nudix family protein (ΦRP12-ORF2 and ΦRP31-ORF1), an RyR domain protein (ΦRP12-ORF45 and ΦRP31-ORF42), a radical SAM domain-containing protein (ΦRP12-ORF76 and ΦRP31-ORF72), an Fe-S oxidoreductase (ΦRP12-ORF77 and ΦRP31-ORF73), a molybdenum cofactor biosynthesis protein A (ΦRP12-ORF79 and ΦRP31-ORF75), a mangotoxin synthesis-involved protein MgoB (ΦRP12-ORF80 and ΦRP31-ORF76), an enoyl-CoA hydratase (ΦRP12-ORF176 and ΦRP31-ORF173), ABC transporter subunits (ΦRP12-ORF191 and –ORF192, and ΦRP31-ORF188 and –ORF189), a TRAP transporter solute receptor like protein (ΦRP12-ORF193 and ΦRP31-ORF190), an XRE family plasmid maintenance system antidote protein (ΦRP12-ORF216 and ΦRP31-ORF268), a Cof hydrolase (ΦRP12-ORF252 and ΦRP31-ORF249), a poly(3-hydroxyalkanoate) depolymerase (ΦRP12-ORF269 and ΦRP31-ORF266), and an N-acetyltransferase (ΦRP12-ORF271 and ΦRP31-ORF268) (Supplementary Table S2).

**Table 1:** β and β′ RNAP-like subunits detected on the phage genomes.

**Virion-associated-RNAP**					**Early-expressed-RNAP**			
	β-subunit (RpoB)^*^		β′-subunit (RpoC)^*^		β-subunit (RpoB)^*^		β′-subunit (RpoC)^*^	
	**N-region**	**C-region**	**N-region**	**C-region**	**N-region+**	**C-region**	**N-region**	**C-region**
φKZ	ORF178	ORF149	ORF180	ORF80	ORF123	ORF71-73	ORF55	ORF74
φRSL2	ORF37	ORF48	ORF38	ORF192	ORF115	ORF209	ORF221	ORF208
φRSF1	ORF40	ORF51	ORF41	ORF199	ORF122	ORF215	ORF227	ORF214
φRP12	ORF41	ORF55	ORF42	ORF258	ORF92	ORF275	ORF287	ORF274
φRP31	ORF38	ORF51	ORF39	ORF255	ORF88	ORF272	ORF285	ORF271

* *Split into two ORFs*.

+ *φKZ ORF123 has been previously annotated as an RpoB fragment (Ceyssens et al., 2014)*.

Four ORFs (ORF1, ORF28, ORF31, and ORF54) of ΦRP12 and two ORFs (ORF103 and ORF283) of ΦRP31 are specific to each phage. There is no information about the actual expression patterns and functions of these gene products during the phage infection cycle.

### ΦRP12 and ΦRP31 infection cycles and effects of rifampicin

The ΦRP12 and ΦRP31 infection cycles were examined using single-step growth experiments with *R. solanacearum* strain MAFF 730138 as the host. Both phages showed almost the same infection patterns and the typical pattern for ΦRP31 is shown in Supplementary Figure S4. One infection cycle took 210 min with a latent period of 90-min. The burst size was approximately 75 plaque-forming units (pfu)/cell. To test if host RNAP is involved in infection of these phages, we analyzed the sensitivity of infection to rifampicin that inhibits RNAP by binding to the β subunits. *R. solanacearum* phages ΦRSL1 and ΦRSB1 were used as controls. ΦRSL1 is a myovirus and does not encode genes for RNAP (Yamada et al., 2010) and ΦRSB1 is a podovirus and encodes a T7-like single peptide RNAP (Kawasaki et al., 2009). The minimal inhibitory concentration (MIC) of rifampicin on strain MAFF 730138 growth was previously shown to be 3 μg/ml (Bhunchoth et al., 2016). The addition of 5 μg/ml rifampicin to bacterial cultures prior (20 min) to ΦRSL1 or ΦRSB1 infection completely abolished progeny phage production. In contrast, ΦRP31-infected cultures produced progeny phages in both the presence and absence of rifampicin even at the concentration as high as 20 μg/ml. ΦRP12 also gave an essentially the same result (data not shown). These observations show that ΦRP12 and ΦRP31 can initiate and complete the infection cycle in the absence of transcription by host RNAP and suggest that phage transcription is carried out by phage encoded RNAPs without the need of the activity of host RNAP. For confirmation, we re-assessed the effects of rifampicin on ΦRSL2 and ΦRSF1 infection. As shown in Table 2, phage development was not detected in the cells treated with rifampicin at 5 μg/ml or higher concentrations for either ΦRSL2 or ΦRSF1.

**Table 2:** Effects of rifampicin (Rif) on the phage amplification.

**Phage**	**Rif (μg/ml)**	**Number of plaques**	
		**×10^4^**	**×10^6^**
φRSB1	0	112 ± 9.71	2 ± 2.00
	5	0	0
	10	0	0
	20	0	0
φRSL1	0	–	556 ± 127
	5	0	0
	10	0	0
	20	0	0
φRP31	0	–	92 ± 8.62
	5	665 ± 39.6	11 ± 2.52
	10	459 ± 26.6	7 ± 3.06
	20	260 ± 37.5	1 ± 0.58
φRSL2	0	1,145 ± 355	15 ± 3.12
	5	0	0
	10	0	0
	20	0	0
φRPSF1	0	2,290 ± 370	19 ± 7.31
	5	0	0
	10	0	0
	20	0	0

*-, Confluent lysis*.

## Discussion

### ΦRP12 and ΦRP31 as ΦKZ-related phages

Several jumbo phages are regarded as ΦKZ-related phages based on their common conserved features (Cornelissen et al., 2012). Morphologically, ΦKZ-related phages have a very large icosahedral head (120–125 nm in diameter) and a long (> 190 nm) contractile tail sometimes associated with fibers (Krylov et al., 2007). Their genomes are large (>200 kbp), circularly permuted, and terminally redundant linear double-stranded (ds) DNA with a G+C content (36–48%) always lower than that of the host (60–88%). Based on genomic and genetic similarity, the ΦKZ-related phages are further subdivided into ΦKZ-like viruses, including ΦKZ, 201Φ2-1, ΦPA3, and EL-like viruses such as EL and OBP (Lavigne et al., 2009; Cornelissen et al., 2012). Both ΦRP12 and ΦRP31 share conserved features of the ΦKZ-related phages but the G+C content is higher in ΦRP12 (53.40%) and ΦRP31 (53.35%). As shown in Figure 2, phylogenetic and comparative analyses at both genomic and gene levels revealed ΦRP12 and ΦRP31 are closely related to previously recognized ΦKZ-related phages, and most closely related to *Ralstonia* phages ΦRSL2 and ΦRSF1 among sequenced phages. Our study also revealed that ΦRP12 and ΦRP31 encode many genes conserved in ΦKZ-like viruses, including the β and β′ subunits of the multisubunit RNAP.

### ΦRP12 and ΦRP31 infection cycles

Host-independent early gene expression mediated by virion-associated-RNAP (the β and β′ subunits of the multisubunit RNAP) was proposed by Ceyssens et al. (2014) in ΦKZ infection. In our previous work, we showed a faster (60 min) and more efficient (1.5-fold larger burst size) infection by ΦRSF1 than ΦRSL2 in the same *Ralstonia* host strain (Bhunchoth et al., 2016). Our proteomic study revealed a full set of β and β′ subunits in ΦRSF1 virions except that a portion of the β′ subunit was undetected in ΦRSL2 virion (Bhunchoth et al., 2016). We could not confirm the involvement of virion-associated-RNAP in early gene expression during phage infection in either of ΦRSF1 or ΦRSL2, because neither ΦRSF1 nor ΦRSL2 could replicate in the host cells treated with rifampicin at MIC levels (3 ~ 5 μg/ml) of host growth (Bhunchoth et al., 2016 and Table 2 in this work). In contrast, ΦRP12 and ΦRP31 could replicate with rifampicin treatment at a concentration of as high as 20 μg/ml, while the same condition completely blocked the replication of ΦRSL1 (a myovirus, Yamada et al., 2010) and ΦRSB1 (a podovirus, Kawasaki et al., 2009) (controls). This clearly showed the ability of ΦRP12 and ΦRP31 to complete their infection in the absence of bacterial RNAP activity, namely actual functioning of both sets of noncanonical multisubunit RNAPs encoded by these phages. Here one question arises as to why similar phages ΦRSL2 and ΦRSF1 cannot replicate in the presence of rifampicin in spite of encoding all of the highly conserved subunit genes as ΦRP12 and ΦRP31 (Table 1). In addition, all of ΦRSL2, ΦRSF1, ΦRP12, and ΦRP31 were found to encode orthologs of ΦKZ gp68, which was found as a fifth subunit of early-expressed-RNAP (Yukunina et al., 2015): ΦRSL2 ORF213 (YP_009213062), ΦRSF1 ORF219 (YP_009208023), ΦRP12 ORF279, and ΦRP31 ORF276. The genomes of ΦRP12 and ΦRP31 are ca. 60 kbp (~25%) larger than those of ΦRSL2 and ΦRSF1. As seen in Figures 1A,B, the extra regions containing approximately 50 ORFs are concentrated in the central part of the ΦRP12 and ΦRP31 genome maps, embedded between large clusters of structural genes. Although most of these ORFs showed no significant homology in the databases, some of them may encode a function involved in the host-independent (or rifampicin-resistant) RNAP activity.

## Author contributions

TY and HO wrote the manuscript. TM (1st author), OC, TK, MF, and TY performed the molecular analysis. GY, TM (3rd author), and HO performed the bioinformatic analysis. TM (1st author) and MN performed the LC-MS/MS analysis. TM (3rd author), GY, TM (1st author), OC, TK, MN, MF, HO, and TY contributed to the concept of this study.

### Conflict of interest statement

The authors declare that the research was conducted in the absence of any commercial or financial relationships that could be construed as a potential conflict of interest.
