# Supplementary material for: Replications of Two Closely Related Groups of Jumbo Phages Show Different Level of Dependence on Host-encoded RNA Polymerase
Source: Front Microbiol. 2017 Jun 13;8:1010. doi: 10.3389/fmicb.2017.01010 (PMC5468394; doi:10.3389/fmicb.2017.01010)
Supplement: Supplementary file 1 [file Table1.PDF]

Table S1. Strains of *R. solanacearum* used in this study.

Strain	Race	Biovar	Phylo type	Host plant	Sensitivity[a] RP12	Sensitivity[a] RP31	Source and Location
C319	1	4	I	Tobacco	+	+	Furuya et el.(1997)[b]
M4S	1	3	I	Tobacco	+	+	JT Inc.[c]
Ps29	1	3	I	Tobacco	+	+	JT Inc.
Ps65	1	3	I	Tobacco	-	-	JT Inc. (Hiroshima)
Ps72	1	4	I	Tobacco	+	+	JT Inc. (Kohchi)
Ps74	1	4	I	Tobacco	+	+	JT Inc. (Yamaguchi)
RS1002	1	4	I	Tomato	+	+	Mukaihara et al., (2004)[d]
MAFF106603	1	3	I	Tomato	+	+	NIAS[e] (Kumamoto)
MAFF106611	1	4	I	Eggplant	+	+	NIAS (Kumamoto)
MAFF211270	1	N2	I	Tomato	-	-	NIAS (Shizuoka)
MAFF211271	3	N2	IV	Potato	-	-	NIAS (Shizuoka)
MAFF211272	4	4	I	Curcuma	-	-	NIAS (Kohchi)
MAFF211514	4	4	I	Tomato	-	-	NIAS (Kohchi)
MAFF301485	1	3	I	Tomato	-	-	NIAS (Ooita)
MAFF301556	1	4	I	Potato	+	+	NIAS (Nagasaki)
MAFF301558	3	N2	I	Potato	-	-	NIAS (Nagasaki)
MAFF327032	3	N2	IV	Potato	+	+	NIAS (Nagasaki)
MAFF730103	1	3	I	Tomato	+	+	NIAS (Gumma)
MAFF730135	1	4	I	Potato	+	+	NIAS (Nagasaki)
MAFF730138	1	3	IV	Tomato	+	+	NIAS (Kohchi)
MAFF730139	1	4	I	Eggplant	+	+	NIAS (Kohchi)

[a]Sensitivity: +, sensitive; -, resistant.
[b]Furuya, N., Yamasaki, S., Nishioka, M., Shiraishi, I., Iiyama, K., and Matsuyama, N. (1997). Antimicrobial activities of *Pseudomonads* against plant pathogenic organisms and efficacy of *Pseudomonas aeruginosa* ATCC7700 against bacterial wilt of tomato. *Annals of Phytopathology Society of Japan*, 65, 417–424.
[c]JT Inc., Japan Tobacco Incorporation, Tokyo, Japan.
[d]Mukaihara, T., Tamura, N., Murata, Y. and Iwabuchi, M. (2004) Genetic screening of Hrp type III-related pathogenicity genes controlled by the HrpB transcriptional activator in *Ralstonia solanacearum*. *Mol Microbiol* 54, 863–875.
[e]NIAS, National Institute of Agrobiological Sciences, Japan.
