# Supplementary material for: Replications of Two Closely Related Groups of Jumbo Phages Show Different Level of Dependence on Host-encoded RNA Polymerase
Source: Front Microbiol. 2017 Jun 13;8:1010. doi: 10.3389/fmicb.2017.01010 (PMC5468394; doi:10.3389/fmicb.2017.01010)
Supplement: Supplementary file 2 [file Table2.pdf]

Table S2A. Annotation of phiRP12 ORFs.

ORF	strand	start	end	length (aa)	Annotation	Uniprot ID	e value
ORF1	-	30	431	133	Hypothetical protein	A4G7B9	3.21E-33
ORF2	-	701	1123	140	MutT/nudix family protein	A0A091ATY7	1.96E-34
ORF3	-	1172	3358	728	T4-like DNA polymerase	A0A158AP48	0
ORF4	-	3460	3906	148	Predicted ORF		
ORF5	-	4344	6161	605	Predicted ORF		
ORF6	-	6163	6570	135	Hypothetical protein	A0A158ARA6	2.47E-32
ORF7	-	6676	6918	80	Predicted ORF		
ORF8	-	6935	7477	180	Predicted ORF		
ORF9	-	7500	7895	131	Predicted ORF		
ORF10	-	7902	8336	144	Predicted ORF		
ORF11	-	8424	8939	171	Predicted ORF		
ORF12	-	8936	9754	272	Predicted ORF		
ORF13	-	9768	10139	123	Predicted ORF		
ORF14	-	10264	11268	334	Hypothetical protein	A0A158ANJ8	1.18E-49
ORF15	-	11357	11872	171	Predicted ORF		
ORF16	+	12249	13052	267	Hypothetical protein	A0A158ANV0	3.97E-99
ORF17	-	13104	13667	187	Predicted ORF		
ORF18	-	13670	14266	198	Predicted ORF		
ORF19	-	14304	14864	186	Predicted ORF		
ORF20	-	15097	15615	172	Predicted ORF		
ORF21	-	16344	16961	205	Predicted ORF		
ORF22	+	17589	18194	201	Predicted ORF		
ORF23	+	18321	19706	461	Hypothetical protein	A0A158ANF9	4.27E-126
ORF24	-	19762	21975	737	Terminase, large subunit	A0A158AN67	0
ORF25	-	22127	23836	569	Virion structural protein	A0A158ANU9	7.04E-115
ORF26	-	23901	26489	862	Putative virion stractural protein	A0A158ANN8	0
ORF27	-	26574	27497	307	Hypothetical protein	A0A158APH4	1.11E-51
ORF28	-	27524	27634	36	Predicted ORF		
ORF29	+	27953	30043	694	Tail shealth protein	A0A158AN48	0
ORF30	+	30119	31039	306	Putative major virion structural protein	A0A158ANS9	7.09E-88
ORF31	-	31163	32815	550	Flagellar hook-length control protein FliK	A0A0C4YEF7	0
ORF32	-	32986	33723	245	Hypothetical protein	R9MDL3	2.65E-07
ORF33	-	33723	34019	98	Predicted ORF		
ORF34	-	34255	34755	166	Predicted ORF		
ORF35	-	34842	35036	64	Predicted ORF		
ORF36	-	35102	35875	257	Predicted ORF		
ORF37	-	36106	36879	257	Putative virion stractural protein	A0A0K2QQL0	2.32E-76
ORF38	-	36886	37608	240	Hypothetical protein	A0A158ANQ2	1.05E-78
ORF39	-	37619	39097	492	Hypothetical protein	A0A158ANE4	3.10E-57
ORF40	-	39101	39295	64	Predicted ORF		
ORF41	-	39394	43845	1483	Putative RNA polymerase beta subunit	A0A158ANL1	0
ORF42	-	43848	45842	664	Putative RNA polymerase beta prime subunit	A0A158ANM3	0
ORF43	+	45960	53462	2500	Glycoside hydrolase family protein	A0A158AMW1	6.54E-89
ORF44	+	53541	55655	704	Hypothetical protein	A0A158ANA8	5.26E-129
ORF45	+	55692	56042	116	RyR domain protein	A0A0A0YVI6	1.37E-43
ORF46	-	56101	57330	409	Hypothetical protein	A0A158ANK2	1.20E-18
ORF47	-	57415	57963	182	Hypothetical protein	A0A158ANA0	5.24E-42
ORF48	-	57988	58833	281	Hypothetical protein	A0A158AQI4	1.49E-08
ORF49	-	58905	59381	158	Predicted ORF		
ORF50	-	59505	59813	102	Predicted ORF		
ORF51	+	60069	60533	154	Predicted ORF		
ORF52	-	60530	61501	323	Hypothetical protein	A0A0K2QR89	1.15E-21
ORF53	-	61449	62099	216	Hypothetical protein	A0A158AP30	2.75E-06
ORF54	-	62096	62902	268	GIY-YIG catalytic domain protein	A0A158ANJ9	1.22E-11
ORF55	-	63018	63692	224	Virion structural protein	A0A158ANL7	8.25E-71
ORF56	-	63711	63998	95	Putative GTP-binding protein	A0A120IEJ7	1.33E-13
ORF57	-	64272	65045	257	Phosphate starvation-inducible protein PhoH	A0A0V7ZT44	6.39E-57
ORF58	-	65190	66041	283	Predicted ORF		
ORF59	-	66111	67517	468	Putative UvsX protein	A0A158ANI6	5.60E-174
ORF60	+	67772	68620	282	Hypothetical protein	A0A158ANA1	9.31E-10
ORF61	-	68702	69106	134	Hypothetical protein	Q8SD08	6.03E-07
ORF62	-	69192	70793	533	Ribonuclease H	A0A158AN34	1.82E-59
ORF63	-	70882	71298	138	Predicted ORF		
ORF64	-	71295	71720	141	Predicted ORF		
ORF65	+	71805	73103	432	Putative virion stractural protein	A0A158AQ83	1.89E-85
ORF66	+	73118	73750	210	Hypothetical protein	A0A158ANE1	1.85E-43
ORF67	-	73802	74740	312	Hypothetical protein	A0A158AMW8	4.55E-53
ORF68	+	74739	74858	39	Predicted ORF		
ORF69	+	74855	77347	830	Putative SbcC-ATPase	A0A158AMV9	9.51E-137
ORF70	-	77389	77943	184	Hypothetical protein	A0A0A8J8X9	3.07E-17
ORF71	-	77964	79220	418	Virion structural protein	A0A158AMX0	2.42E-79
ORF72	+	79417	81021	534	YomR	A0A158AMM8	1.68E-109
ORF73	+	81074	82351	425	YomR	W8CZP9	1.66E-94
ORF74	+	82427	82804	125	Predicted ORF		
ORF75	+	83085	83972	295	Predicted ORF		
ORF76	+	84042	85655	537	Radical SAM domain-containing protein	U2PWE8	3.22E-14
ORF77	+	85669	86751	360	Arylsulfatase regulator (Fe-S oxidoreductase)	G4Q4C0	7.56E-07
ORF78	+	86748	87896	382	Predicted ORF		
ORF79	+	87893	89152	419	Molybdenum cofactor biosynthesis protein A	A0A0P1F1H8	1.04E-29
ORF80	+	89161	89997	278	Mangotoxin biosynthesis-involved protein MgoB	A0A0X1T461	9.30E-62
ORF81	-	90048	90695	215	Predicted ORF		
ORF82	-	90860	91504	214	Hypothetical protein	A0A0A8J9A2	1.92E-21
ORF83	-	91577	91858	93	Predicted ORF		
ORF84	-	91909	92247	112	Predicted ORF		
ORF85	-	92263	92727	154	Predicted ORF		
ORF86	-	92802	93395	197	Crossover junction endodeoxyribonuclease RuvC	A0A158AMN9	6.02E-35
ORF87	-	93404	94288	294	Virion structural protein	A0A158APY6	3.92E-69
ORF88	-	94367	96613	748	Virion structural protein	A0A158AM86	5.09E-140
ORF89	+	96702	99506	934	Putative virion stractural protein	A0A158AMM1	0
ORF90	+	99503	99727	74	Predicted ORF		
ORF91	-	99946	102066	706	Predicted ORF		
ORF92	-	102145	103821	558	Putative RNA polymerase beta subunit	A0A158AMP5	2.48E-135
ORF93	-	103874	105028	384	Hypothetical protein	A0A158APR4	5.27E-08

ORF94	-	105311	107470	719	Putative major head protein	A0A158AMG1	6.61E-174
ORF95	-	107520	108083	187	Hypothetical protein	A0A158AM88	1.82E-06
ORF96	+	108308	109843	511	Putative DnaB helicase	A0A158AMU7	1.58E-165
ORF97	-	109903	110694	263	Hypothetical protein	A0A0C5K996	2.58E-45
ORF98	-	110834	111409	191	Predicted ORF		
ORF99	-	111500	111847	115	Hypothetical protein	A0A0D0HCC4	3.72E-23
ORF100	-	111926	112351	141	Hypothetical protein	Q2Z0W1	1.13E-08
ORF101	-	112410	112610	66	Predicted ORF		
ORF102	-	112623	112748	41	Predicted ORF		
ORF103	-	112720	113088	122	Hypothetical protein	A0A0D0FRY7	9.52E-48
ORF104	-	113160	113957	265	Predicted ORF		
ORF105	-	114019	115017	332	Predicted ORF		
ORF106	-	115209	116135	308	Predicted ORF		
ORF107	-	117075	117503	142	Predicted ORF		
ORF108	-	117544	117873	109	Predicted ORF		
ORF109	-	118034	118384	116	Predicted ORF		
ORF110	-	118447	118707	86	Predicted ORF		
ORF111	-	118801	119127	108	Predicted ORF		
ORF112	-	119143	119283	46	Predicted ORF		
ORF113	-	119300	119632	110	Predicted ORF		
ORF114	-	119643	120020	125	Predicted ORF		
ORF115	-	120096	120527	143	Predicted ORF		
ORF116	-	120743	121546	267	Predicted ORF		
ORF117	-	121543	122541	332	Hypothetical protein		
ORF118	-	122620	123525	301	Predicted ORF		
ORF119	-	123647	124054	135	Predicted ORF		
ORF120	-	124171	124833	220	Predicted ORF		
ORF121	-	124936	125292	118	Predicted ORF		
ORF122	-	125367	125756	129	Predicted ORF		
ORF123	-	125784	127139	451	Mitochondrial chaperone BCS1	A0A0W4ZVQ0	1.25E-20
ORF124	-	127238	127846	202	Predicted ORF		
ORF125	-	127867	128502	211	Hypothetical protein	W6ARW1	3.96E-27
ORF126	-	128615	128965	116	Hypothetical protein	Q7NXS4	5.22E-36
ORF127	-	129013	129648	211	Predicted ORF		
ORF128	-	130566	130691	41	Predicted ORF		
ORF129	-	130688	130879	63	Predicted ORF		
ORF130	-	130876	131106	76	Hypothetical protein	A0A158E9B3	1.04E-10
ORF131	-	131103	131555	150	Hypothetical protein	A0A106QCE2	2.08E-46
ORF132	-	132543	133418	291	Predicted ORF		
ORF133	-	133559	133909	116	Predicted ORF		
ORF134	-	134001	134603	200	Deoxycytidine triphosphate deaminase	A0A0Q5FHS0	6.79E-58
ORF135	-	134647	134781	44	Predicted ORF		
ORF136	-	135049	136851	600	Hypothetical protein	F8SJ78	1.23E-86
ORF137	-	136862	137218	118	Predicted ORF		
ORF138	-	137337	138377	346	Hypothetical protein	H8ZN24	8.52E-42
ORF139	-	138370	139410	346	Hypothetical protein	E3SQ11	1.31E-71
ORF140	-	139411	140058	215	Hypothetical protein	M4PM32	1.19E-09
ORF141	-	140073	140549	158	Predicted ORF		
ORF142	-	140550	141428	292	Predicted ORF		
ORF143	-	141488	142000	170	Hypothetical protein	A0A081BFQ3	9.02E-09
ORF144	-	142149	142757	202	Predicted ORF		
ORF145	-	143074	143316	80	Predicted ORF		
ORF146	-	143433	143615	60	Predicted ORF		
ORF147	-	143928	144251	107	Predicted ORF		
ORF148	-	144252	145937	561	Hypothetical protein	M1LRP3	1.72E-06
ORF149	-	146259	147920	553	Predicted ORF		
ORF150	-	148019	148435	138	Hypothetical protein	C6XE84	5.72E-09
ORF151	-	148490	148771	93	Predicted ORF		
ORF152	-	148768	150426	552	Predicted ORF		
ORF153	-	150426	153695	1089	Putative tail fiber protein 1	M1F173	4.60E-170
ORF154	-	153709	154257	182	Hypothetical protein	A0A0A8J8X9	5.38E-07
ORF155	-	154413	155615	400	Ribonucleotide reductase of class Ia (Aerobic), beta subunit	W8X2K7	2.51E-116
ORF156	-	155753	158413	886	Ribonucleoside-diphosphate reductase	I7CD23	0
ORF157	-	158703	159158	151	Predicted ORF		
ORF158	-	159155	159736	193	Predicted ORF		
ORF159	-	159736	160386	216	Predicted ORF		
ORF160	-	160395	160841	148	Predicted ORF		
ORF161	-	161014	162975	653	DNA ligase	A0A0J1JV68	6.86E-119
ORF162	-	162972	163679	235	Predicted ORF		
ORF163	-	163772	164035	87	Predicted ORF		
ORF164	-	164100	164618	172	Predicted ORF		
ORF165	-	164688	166517	609	Hypothetical protein	K4ZBQ3	1.60E-07
ORF166	-	166687	167346	219	Transglycosylase	A0A125PA11	6.81E-21
ORF167	-	167507	167890	127	Hypothetical protein	C1A414	1.08E-35
ORF168	-	168100	169110	336	Phosphoesterase	U9U3G0	9.91E-27
ORF169	-	169200	171455	751	DEAD-like helicase	W6AS32	1.07E-157
ORF170	-	171584	172123	179	Hypothetical protein	K7HKD1	1.99E-06
ORF171	-	172141	172575	144	Predicted ORF		
ORF172	-	172612	172992	126	Hypothetical protein	A0A0Q4NJE4	2.87E-38
ORF173	-	173000	173350	116	Predicted ORF		
ORF174	-	173347	173583	78	Predicted ORF		
ORF175	-	173587	173907	106	Predicted ORF		
ORF176	+	174091	174534	147	Enoyl-CoA hydratase	A0A0Q7FG53	4.79E-36
ORF177	-	174599	175039	146	Predicted ORF		
ORF178	-	175116	175592	158	Predicted ORF		
ORF179	-	175636	176172	178	Predicted ORF		
ORF180	-	176325	176840	171	Predicted ORF		
ORF181	-	176840	177706	288	Predicted ORF		
ORF182	-	177827	178171	114	Predicted ORF		
ORF183	-	178313	179194	293	Hypothetical protein	A0A0D7KF63	2.51E-07
ORF184	-	179293	179616	107	Predicted ORF		
ORF185	-	179699	180223	174	Dihydrofolate reductase	A0A059X418	1.20E-33
ORF186	-	180266	180895	209	Hypothetical protein	C4MZR9	6.64E-08
ORF187	-	180895	181602	235	Predicted ORF		
ORF188	-	181662	182966	434	Predicted ORF		

ORF189	-	183099	184181	360	Predicted ORF		
ORF190	-	184247	184780	177	Predicted ORF		
ORF191	-	184901	185638	245	ABC-type transporter, integral membrane subunit	F2LVG9	6.73E-18
ORF192	-	185638	186426	262	ABC transporter substrate-binding protein	A0A0U3E8W3	4.59E-14
ORF193	-	186498	187418	306	TRAP transporter solute receptor like protein	B2ZXX0	3.79E-73
ORF194	-	187453	187596	47	Predicted ORF		
ORF195	-	187593	188384	263	Hypothetical protein	A0A0X3AKY0	1.23E-24
ORF196	-	188502	188984	160	Predicted ORF		
ORF197	-	188996	189352	118	Predicted ORF		
ORF198	-	189364	189714	116	Hypothetical protein	F5JC11	4.23E-31
ORF199	-	189707	189952	81	Predicted ORF		
ORF200	+	190017	190271	84	Hypothetical protein	A0A0A8J8D1	4.78E-25
ORF201	-	190311	190766	151	Predicted ORF		
ORF202	-	190773	191057	94	Predicted ORF		
ORF203	+	191161	192888	575	Hypothetical protein	G0MXP9	5.16E-08
ORF204	-	192957	193646	229	Predicted ORF		
ORF205	-	193657	194427	256	Predicted ORF		
ORF206	-	194494	195861	455	ATPase associated with various cellular activities family protein	I7DKJ3	5.82E-22
ORF207	-	196007	196429	140	Predicted ORF		
ORF208	-	196429	196755	108	Predicted ORF		
ORF209	-	196759	198051	430	Putative RNA ligase	F8SJC5	8.55E-57
ORF210	-	198057	199187	376	Hypothetical protein	K4K2V0	5.76E-17
ORF211	-	199238	201004	588	Nicotinate phosphoribosyltransferase	A0A098UDR3	9.45E-153
ORF212	-	201043	201957	304	Ribose-phosphate pyrophosphokinase	K9Z5A2	2.49E-60
ORF213	-	201963	203231	422	Putative RtcB-like protein	W6AR47	0
ORF214	-	203312	204001	229	Predicted ORF		
ORF215	-	204098	205000	300	Thymidylate synthase	S7HVU0	1.35E-116
ORF216	-	205091	205441	116	XRE family plasmid maintenance system antidote protein	A0A158E9F6	1.33E-06
ORF217	-	205444	205896	150	Predicted ORF		
ORF218	-	205907	206287	126	Predicted ORF		
ORF219	-	206291	206557	88	Predicted ORF		
ORF220	-	206632	207300	222	Predicted ORF		
ORF221	-	207481	208143	220	Thymidylate kinase	F1ZRR9	3.05E-25
ORF222	-	208207	208473	88	Predicted ORF		
ORF223	-	208475	208696	73	Predicted ORF		
ORF224	-	208778	209125	115	Predicted ORF		
ORF225	-	209155	209562	135	Hypothetical protein	A0A158CML1	1.90E-09
ORF226	-	209751	209912	53	Predicted ORF		
ORF227	-	209909	210091	60	Predicted ORF		
ORF228	-	210094	210315	73	Predicted ORF		
ORF229	-	210658	211041	127	Predicted ORF		
ORF230	-	211208	211516	102	Predicted ORF		
ORF231	-	211658	213019	453	Virion structural protein	A0A158CN86	3.86E-89
ORF232	-	213020	213697	225	Hypothetical protein	A0A158CN53	3.54E-27
ORF233	-	213748	215109	453	Putative virion stractural protein	A0A158CN49	1.65E-113
ORF234	-	215119	216702	526	Hypothetical protein	A0A158CMZ4	1.09E-71
ORF235	-	216837	218393	518	Predicted ORF		
ORF236	-	218395	219594	399	Predicted ORF		
ORF237	-	219681	220982	433	Predicted ORF		
ORF238	-	220992	222431	479	Predicted ORF		
ORF239	-	222604	224028	474	Predicted ORF		
ORF240	-	224120	225448	442	Predicted ORF		
ORF241	-	225524	227299	591	Predicted ORF		
ORF242	-	227443	228042	199	Virion structural protein	A0A158CPM5	1.02E-23
ORF243	-	228046	228942	298	Putative virion structural protein	A0A158CNH4	8.81E-32
ORF244	-	229024	230319	431	Virion structural protein	A0A158CNF7	9.93E-72
ORF245	+	230350	231507	385	Virion structural protein	A0A158CNJ1	1.92E-99
ORF246	+	231519	234446	975	Putative virion stractural protein	A0A158CNF2	0
ORF247	-	234505	235941	478	Predicted ORF		
ORF248	-	236024	237358	444	Predicted ORF		
ORF249	-	237369	237821	150	Hypothetical protein	A0A158CNM9	2.21E-11
ORF250	-	237882	239183	433	Virion structural protein	A0A158CNH2	3.72E-31
ORF251	+	239369	241252	627	T4-like DNA polymerase	A0A158CQK8	0
ORF252	+	241324	241821	165	Cof hydrolase	A0A0K0PVE7	1.08E-38
ORF253	-	241880	242242	120	Nuclease (SNase domain-containing protein)	A0A0H4BVY0	6.27E-19
ORF254	-	242783	243223	146	Hypothetical protein	A0A158CNR1	5.64E-09
ORF255	-	243223	244869	548	Hypothetical protein	A0A158CNU8	3.07E-92
ORF256	-	244883	245674	263	Hypothetical protein	A0A158CNW9	3.47E-11
ORF257	+	245854	246732	292	Predicted ORF		
ORF258	+	246742	248070	442	Putative RNA polymerase beta prime subunit	A0A158CNJ7	4.23E-152
ORF259	-	248460	249107	215	Hypothetical protein	A0A0S4NSA1	8.71E-92
ORF260	-	249393	249692	99	Predicted ORF		
ORF261	+	249996	250220	74	Predicted ORF		
ORF262	+	250316	250675	119	Hypothetical protein	B5ZX55	2.02E-17
ORF263	+	250786	251256	156	Hypothetical protein	R4KMJ1	7.45E-07
ORF264	+	251330	252106	258	Hypothetical protein	A0A158CNH8	3.52E-22
ORF265	+	252198	253043	281	Putative peptidoglycan binding domain protein	A0A0A8J8S6	6.06E-50
ORF266	-	253111	254046	311	Predicted ORF		
ORF267	-	254112	255596	494	RAD2/SF2 helicase	A0A158APK7	3.84E-80
ORF268	-	255707	256366	219	Hypothetical protein		
ORF269	+	256412	257227	271	Poly(3-hydroxyalkanoate) depolymerase	A0A069PPN5	2.65E-117
ORF270	-	257298	257732	144	Predicted ORF		
ORF271	-	257768	258202	144	Putative N-acetyltransferase	A0A0K2QQJ6	4.29E-42
ORF272	-	258256	258666	136	Hypothetical protein	A0A0Q4N9E9	8.27E-06
ORF273	-	258632	258937	101	Hypothetical protein	A0A158EGG2	1.28E-15
ORF274	-	258990	261032	680	Putative RNA polymerase beta prime subunit	A0A158AQG9	5.55E-177
ORF275	-	261090	263393	767	DNA-directed RNA polymerase subunit beta	A0A158APS3	0
ORF276	+	263582	263926	114	Hypothetical protein	A0A158API7	6.11E-08
ORF277	-	264017	265759	580	Hypothetical protein	A0A158ARQ9	8.94E-38
ORF278	-	265821	267524	567	Hypothetical protein	A0A158APW8	5.97E-11
ORF279	-	267591	269312	573	Hypothetical protein	A0A0K2QRM1	5.02E-31
ORF280	-	269483	270205	240	Hypothetical protein	A0A158APF8	4.98E-15
ORF281	-	270274	271104	276	Hypothetical protein	A0A158AQB4	6.42E-57
ORF282	-	271108	272313	401	Nuclease SbcCD, D subunit	A0A158APM3	6.16E-103
ORF283	-	272297	272875	192	Hypothetical protein	A0A0A8JBH1	1.46E-10

ORF284	-	272957	273535	192	Predicted ORF		
ORF285	-	273528	273932	134	Hypothetical protein	A0A158APS2	9.50E-21
ORF286	-	274041	274664	207	Hypothetical protein	A0A158AP93	2.11E-26
ORF287	-	274706	276223	505	DNA-directed RNA polymerase subunit beta	A0A158APA7	7.06E-134
ORF288	-	276341	278416	691	Hypothetical protein	A0A158APF9	3.82E-47
ORF289	+	278754	279836	360	Hypothetical protein	A0A158AP78	2.92E-68

Table S2B. Annotation of phiRP31 ORFs.

ORF	strand	start	end	length (aa)	Annotation	Uniprot ID	e value
ORF1	-	30	452	140	MutT/nudix family protein	Q5QW66	1.39E-33
ORF2	-	501	2687	728	T4-like DNA polymerase	A0A158AP48	0
ORF3	-	2789	3235	148	Predicted ORF		
ORF4	-	3673	5490	605	Predicted ORF		
ORF5	-	5492	5899	135	Hypothetical protein	A0A158ARA6	2.79E-32
ORF6	-	6009	6251	80	Predicted ORF		
ORF7	-	6268	6810	180	Predicted ORF		
ORF8	-	6833	7228	131	Predicted ORF		
ORF9	-	7235	7669	144	Predicted ORF		
ORF10	-	7757	8272	171	Predicted ORF		
ORF11	-	8269	9087	272	Predicted ORF		
ORF12	-	9101	9472	123	Predicted ORF		
ORF13	-	9598	10602	334	Hypothetical protein	A0A158ANJ8	4.46E-50
ORF14	-	10691	11170	159	Predicted ORF		
ORF15	+	11556	12359	267	Hypothetical protein	A0A158ANV0	4.53E-99
ORF16	-	12411	12974	187	Predicted ORF		
ORF17	-	12977	13534	185	Predicted ORF		
ORF18	-	13612	14172	186	Predicted ORF		
ORF19	-	14485	15006	173	Predicted ORF		
ORF20	-	15735	16352	205	Predicted ORF		
ORF21	+	16980	17585	201	Predicted ORF		
ORF22	+	17714	18979	421	Hypothetical protein	A0A158ANF9	4.60E-127
ORF23	-	19035	21248	737	Terminase, large subunit	A0A158AN67	0
ORF24	-	21400	23109	569	Virion structural protein	A0A158ANU9	1.48E-115
ORF25	-	23173	25761	862	Putative virion stractural protein	A0A158ANN8	0
ORF26	-	25846	26769	307	Hypothetical protein	A0A158APH4	9.54E-52
ORF27	+	27225	29315	696	Putative tail sheath	A0A158AN48	0
ORF28	+	29391	30311	306	Putative major virion structural protein	A0A158ANS9	9.11E-88
ORF29	-	30434	31171	245	Hypothetical protein	R9MDL3	9.45E-08
ORF30	-	31174	31470	98	Predicted ORF		
ORF31	-	31706	32209	167	Predicted ORF		
ORF32	-	32297	32491	64	Predicted ORF		
ORF33	-	32557	33330	257	Predicted ORF		
ORF34	-	33561	34334	257	Putative virion stractural protein	A0A0K2QQL0	2.17E-76
ORF35	-	34341	35063	240	Hypothetical protein	A0A158ANQ2	1.05E-78
ORF36	-	35074	36552	492	Hypothetical protein	A0A158ANE4	2.85E-57
ORF37	-	36556	36729	57	Predicted ORF		
ORF38	-	36849	41297	1482	Putative RNA polymerase beta subunit	A0A158ANL1	0
ORF39	-	41300	43294	664	Putative RNA polymerase beta prime subunit	A0A158ANM3	0
ORF40	+	43412	50920	2502	Putative soluble lytic murein transglycosylase	A0A0A8J8P3	3.51E-56
ORF41	+	50999	53113	704	Hypothetical protein	A0A158ANA8	1.24E-128
ORF42	+	53149	53499	116	RyR domain protein	A0A0A0YVI6	1.68E-43
ORF43	-	53558	54787	409	Hypothetical protein	A0A158ANK2	2.20E-18
ORF44	-	54872	55420	182	Hypothetical protein	A0A158ANA0	1.21E-42
ORF45	-	55445	56290	281	Hypothetical protein	A0A158AQI4	1.47E-08
ORF46	-	56362	56838	158	Predicted ORF		
ORF47	-	56962	57369	135	Predicted ORF		
ORF48	+	57472	57990	172	Predicted ORF		
ORF49	-	57987	58958	323	Hypothetical protein	A0A0K2QR89	1.13E-21
ORF50	-	58906	59556	216	Predicted ORF		
ORF51	-	59558	60232	224	Virion structural protein	A0A158ANL7	9.71E-71
ORF52	-	60251	60538	95	Putative GTP-binding protein	I4IU58	2.37E-12
ORF53	-	60812	61585	257	Phosphate starvation-inducible protein PhoH	A0A0V7ZT44	6.39E-57
ORF54	-	61899	62786	295	Predicted ORF		
ORF55	-	62856	64262	468	Putative UvsX protein	A0A158ANI6	5.60E-174
ORF56	+	64550	65365	271	Hypothetical protein	A0A158ANA1	9.99E-10
ORF57	-	65447	65797	116	Hypothetical protein	Q8SD08	3.76E-07
ORF58	-	65936	67537	533	Ribonuclease H	A0A158AN34	5.46E-59
ORF59	-	67627	68043	138	Predicted ORF		
ORF60	-	68040	68465	141	Predicted ORF		
ORF61	+	68550	69848	432	Putative virion structural protein	A0A158AQ83	1.43E-85
ORF62	+	69863	70495	210	Hypothetical protein	A0A158ANE1	1.85E-43
ORF63	-	70547	71485	312	Hypothetical protein	A0A158AMW8	4.55E-53
ORF64	+	71484	71603	39	Predicted ORF		
ORF65	+	71600	74092	830	Putative SbcC-ATPase	A0A158AMV9	9.51E-137
ORF66	-	74134	74688	184	Hypothetical protein	A0A0A8J8X9	3.07E-17
ORF67	-	74709	75965	418	Virion structural protein	A0A158AMX0	8.33E-79
ORF68	+	76162	77766	534	YomR	A0A158AMM8	1.68E-109
ORF69	+	77819	79096	425	YomR	W8CZP9	1.66E-94
ORF70	+	79172	79549	125	Predicted ORF		
ORF71	+	79830	80717	295	Predicted ORF		
ORF72	+	80787	82400	537	Radical SAM domain-containing protein	U2PWE8	3.22E-14
ORF73	+	82414	83496	360	Arylsulfatase regulator (Fe-S oxidoreductase)	G4Q4C0	7.56E-07
ORF74	+	83493	84641	382	Predicted ORF		
ORF75	+	84638	85897	419	Molybdenum cofactor biosynthesis protein A	A0A0P1F1H8	1.04E-29
ORF76	+	85906	86742	278	Mangotoxin biosynthesis-involved protein MgoB	A0A0X1T461	9.30E-62
ORF77	-	86793	87440	215	Predicted ORF		
ORF78	-	87605	88219	204	Hypothetical protein	A0A0A8J9A2	1.04E-21
ORF79	-	88322	88603	93	Predicted ORF		
ORF80	-	88654	88992	112	Predicted ORF		
ORF81	-	89008	89472	154	Predicted ORF		
ORF82	-	89546	90139	197	Crossover junction endodeoxyribonuclease RuvC	A0A158AMN9	6.02E-35
ORF83	-	90148	91032	294	Virion structural protein	A0A158APY6	3.92E-69
ORF84	-	91111	93357	748	Putative virion structural protein	A0A158AM86	6.65E-140
ORF85	+	93446	96250	934	Putative virion structural protein	A0A158AMM1	0
ORF86	+	96247	96471	74	Predicted ORF		
ORF87	-	96692	98812	706	Predicted ORF		
ORF88	-	98891	100567	558	Putative RNA polymerase beta subunit	A0A158AMP5	2.48E-135
ORF89	-	100620	101774	384	Hypothetical protein	A0A158APR4	5.27E-08
ORF90	-	102057	104216	719	Putative major head protein	A0A158AMG1	6.61E-174
ORF91	-	104266	104829	187	Hypothetical protein	A0A158AM88	1.82E-06
ORF92	+	105054	106589	511	Putative DnaB helicase	A0A158AMU7	1.58E-165
ORF93	-	106650	107441	263	Hypothetical protein	A0A0C5K996	2.58E-45
ORF94	-	107581	108156	191	Predicted ORF		
ORF95	-	108247	108594	115	Hypothetical protein	A0A0D0HCC4	3.72E-23
ORF96	-	108673	109098	141	Hypothetical protein	Q2Z0W1	1.13E-08
ORF97	-	109157	109357	66	Predicted ORF		
ORF98	+	109356	109466	36	Predicted ORF		

ORF99	-	109467	109844	125	Hypothetical protein	A0A0D0FRY7	9.60E-48
ORF100	-	109907	110704	265	Predicted ORF		
ORF101	-	110766	111764	332	Predicted ORF		
ORF102	-	111956	112882	308	Predicted ORF		
ORF103	+	113047	113334	95	Predicted ORF		
ORF104	-	113822	114250	142	Predicted ORF		
ORF105	-	114291	114626	111	Predicted ORF		
ORF106	-	114781	115131	116	Predicted ORF		
ORF107	-	115194	115454	86	Predicted ORF		
ORF108	-	115548	115874	108	Predicted ORF		
ORF109	-	115890	116030	46	Predicted ORF		
ORF110	-	116047	116379	110	Predicted ORF		
ORF111	-	116390	116767	125	Predicted ORF		
ORF112	-	116843	117274	143	Predicted ORF		
ORF113	-	117490	118293	267	Predicted ORF		
ORF114	-	118290	119288	332	Hypothetical protein		
ORF115	-	119367	120272	301	Predicted ORF		
ORF116	-	120394	120750	118	Predicted ORF		
ORF117	-	120918	121580	220	Predicted ORF		
ORF118	-	121683	122039	118	Predicted ORF		
ORF119	-	122114	122503	129	Predicted ORF		
ORF120	-	122531	123886	451	Mitochondrial chaperone BCS1	A0A0W4ZVQ0	1.25E-20
ORF121	-	123984	124592	202	Predicted ORF		
ORF122	-	124613	125248	211	Hypothetical protein	W6ARW1	3.96E-27
ORF123	-	125361	125711	116	Hypothetical protein	Q7NXS4	5.22E-36
ORF124	-	125759	126394	211	Predicted ORF		
ORF125	-	127312	127437	41	Predicted ORF		
ORF126	-	127434	127625	63	Predicted ORF		
ORF127	-	127622	127852	76	Hypothetical protein	A0A158E9B3	1.04E-10
ORF128	-	127849	128301	150	Hypothetical protein	A0A106QCE2	2.08E-46
ORF129	-	129289	130164	291	Predicted ORF		
ORF130	-	130305	130655	116	Predicted ORF		
ORF131	-	130747	131349	200	Deoxycytidine triphosphate deaminase	A0A0Q5FHS0	6.79E-58
ORF132	-	131393	131527	44	Predicted ORF		
ORF133	-	131795	133597	600	Hypothetical protein	F8SJ78	1.23E-86
ORF134	-	133608	133964	118	Predicted ORF		
ORF135	-	134083	135123	346	Hypothetical protein	H8ZN24	8.52E-42
ORF136	-	135116	136156	346	Hypothetical protein	E3SQ11	1.66E-71
ORF137	-	136157	136768	203	Hypothetical protein	M4PM32	1.18E-09
ORF138	-	136819	137295	158	Predicted ORF		
ORF139	-	137296	138174	292	Predicted ORF		
ORF140	-	138234	138746	170	Hypothetical protein	A0A081BFQ3	9.02E-09
ORF141	-	138895	139503	202	Predicted ORF		
ORF142	-	139820	140062	80	Predicted ORF		
ORF143	-	140179	140361	60	Predicted ORF		
ORF144	-	140674	140997	107	Predicted ORF		
ORF145	-	140998	142683	561	Hypothetical protein	M1LRP3	1.72E-06
ORF146	-	143005	144702	565	Predicted ORF		
ORF147	-	144765	145181	138	Hypothetical protein	C6XE84	5.72E-09
ORF148	-	145236	145457	73	Predicted ORF		
ORF149	-	145514	147172	552	Predicted ORF		
ORF150	-	147172	150441	1089	Putative tail fiber protein 1	M1F173	4.60E-170
ORF151	-	150455	151003	182	Hypothetical protein	A0A0A8J8X9	5.38E-07
ORF152	-	151159	152361	400	Ribonucleotide reductase of class Ia (Aerobic), beta subunit	W8X2K7	2.51E-116
ORF153	-	152499	155159	886	Ribonucleoside-diphosphate reductase	I7CD23	0
ORF154	-	155449	155904	151	Predicted ORF		
ORF155	-	155901	156482	193	Predicted ORF		
ORF156	-	156482	157132	216	Predicted ORF		
ORF157	-	157141	157587	148	Predicted ORF		
ORF158	-	157760	159721	653	DNA ligase	A0A0J1JV68	8.78E-119
ORF159	-	159718	160425	235	Predicted ORF		
ORF160	-	160518	160781	87	Predicted ORF		
ORF161	-	160846	161364	172	Predicted ORF		
ORF162	-	161434	163263	609	Hypothetical protein	K4ZBQ3	1.60E-07
ORF163	-	163433	164092	219	Transglycosylase	A0A125PA11	6.81E-21
ORF164	-	164253	164636	127	Hypothetical protein	C1A414	1.08E-35
ORF165	-	164846	165856	336	Phosphoesterase	U9U3G0	9.91E-27
ORF166	-	165946	168201	751	DEAD-like helicase	W6AS32	1.07E-157
ORF167	-	168330	168869	179	Hypothetical protein	K7HKD1	1.99E-06
ORF168	-	168887	169321	144	Predicted ORF		
ORF169	-	169358	169738	126	Hypothetical protein	A0A0Q4NJE4	2.87E-38
ORF170	-	169746	170096	116	Predicted ORF		
ORF171	-	170093	170329	78	Predicted ORF		
ORF172	-	170333	170653	106	Predicted ORF		
ORF173	+	170837	171280	147	Enoyl-CoA hydratase	A0A0Q7FG53	4.79E-36
ORF174	-	171345	171785	146	Predicted ORF		
ORF175	-	171862	172338	158	Predicted ORF		
ORF176	-	172382	172918	178	Predicted ORF		
ORF177	-	173071	173586	171	Predicted ORF		
ORF178	-	173586	174479	297	Predicted ORF		
ORF179	-	174573	174917	114	Predicted ORF		
ORF180	-	175059	175940	293	Hypothetical protein	A0A0D7KF63	2.51E-07
ORF181	-	176039	176362	107	Predicted ORF		
ORF182	-	176445	176969	174	Dihydrofolate reductase	A0A059X418	1.20E-33
ORF183	-	177012	177641	209	Hypothetical protein	C4MZR9	6.64E-08
ORF184	-	177641	178348	235	Predicted ORF		
ORF185	-	178408	179712	434	Predicted ORF		
ORF186	-	179845	180927	360	Predicted ORF		
ORF187	-	180993	181526	177	Predicted ORF		
ORF188	-	181647	182384	245	ABC-type transporter, integral membrane subunit	F2LVG9	6.73E-18
ORF189	-	182384	183172	262	ABC transporter substrate-binding protein	A0A0U3E8W3	4.59E-14
ORF190	-	183244	184164	306	TRAP transporter solute receptor like protein	B2ZXX0	3.79E-73
ORF191	-	184199	184342	47	Predicted ORF		
ORF192	-	184339	185130	263	Hypothetical protein	A0A0X3AKY0	1.23E-24
ORF193	-	185248	185730	160	Predicted ORF		
ORF194	-	185742	186098	118	Predicted ORF		
ORF195	-	186110	186460	116	Hypothetical protein	F5JC11	4.23E-31
ORF196	-	186453	186698	81	Predicted ORF		
ORF197	+	186763	187017	84	Hypothetical protein	A0A0A8J8D1	4.78E-25
ORF198	-	187057	187398	113	Predicted ORF		

ORF199	-	187519	187803	94	Predicted ORF		
ORF200	+	187907	189634	575	Hypothetical protein	G0MXP9	5.16E-08
ORF201	-	189703	190392	229	Predicted ORF		
ORF202	-	190403	191173	256	Predicted ORF		
ORF203	-	191240	192607	455	ATPase associated with various cellular activities family protein	I7DKJ3	5.82E-22
ORF204	-	192753	193178	141	Predicted ORF		
ORF205	-	193175	193501	108	Predicted ORF		
ORF206	-	193505	194797	430	Putative RNA ligase	F8SJC5	8.55E-57
ORF207	-	194803	195933	376	Hypothetical protein	K4K2V0	5.76E-17
ORF208	-	195984	197750	588	Nicotinate phosphoribosyltransferase	A0A098UDR3	9.45E-153
ORF209	-	197789	198703	304	Ribose-phosphate pyrophosphokinase	K9Z5A2	2.49E-60
ORF210	-	198709	199977	422	Putative RtcB-like protein	W6AR47	0
ORF211	-	200060	200749	229	Predicted ORF		
ORF212	-	200846	201748	300	Thymidylate synthase	S7HVU0	1.35E-116
ORF213	-	201839	202189	116	XRE family plasmid maintenance system antidote protein	A0A158E9F6	1.33E-06
ORF214	-	202192	202644	150	Predicted ORF		
ORF215	-	202655	203035	126	Predicted ORF		
ORF216	-	203039	203305	88	Predicted ORF		
ORF217	-	203380	204048	222	Predicted ORF		
ORF218	-	204233	204862	209	Thymidylate kinase	F1ZRR9	1.67E-25
ORF219	-	204959	205225	88	Predicted ORF		
ORF220	-	205227	205448	73	Predicted ORF		
ORF221	-	205530	205877	115	Predicted ORF		
ORF222	-	205907	206314	135	Hypothetical protein	A0A158CML1	1.90E-09
ORF223	-	206503	206664	53	Predicted ORF		
ORF224	-	206661	206843	60	Predicted ORF		
ORF225	-	206846	207067	73	Predicted ORF		
ORF226	-	207410	207793	127	Predicted ORF		
ORF227	-	207960	208268	102	Predicted ORF		
ORF228	-	208410	209771	453	Virion structural protein	A0A158CN86	3.86E-89
ORF229	-	209772	210449	225	Hypothetical protein	A0A158CN53	3.54E-27
ORF230	-	210500	211861	453	Putative virion stractural protein	A0A158CN49	1.65E-113
ORF231	-	211871	213454	527	Hypothetical protein	A0A158CMZ4	4.76E-71
ORF232	-	213590	215146	518	Predicted ORF		
ORF233	-	215148	216320	390	Predicted ORF		
ORF234	-	216434	217735	433	Predicted ORF		
ORF235	-	217745	219184	479	Predicted ORF		
ORF236	-	219357	220781	474	Predicted ORF		
ORF237	-	220873	222177	434	Predicted ORF		
ORF238	-	222277	224052	591	Predicted ORF		
ORF239	-	224196	224795	199	Virion structural protein	A0A158CPM5	1.02E-23
ORF240	-	224799	225695	298	Putative virion structural protein	A0A158CNH4	8.81E-32
ORF241	-	225777	227045	422	Virion structural protein	A0A158CNF7	9.41E-72
ORF242	+	227103	228260	385	Virion structural protein	A0A158CNJ1	1.92E-99
ORF243	+	228272	231199	975	Putative virion stractural protein	A0A158CNF2	0
ORF244	-	231258	232694	478	Predicted ORF		
ORF245	-	232777	234111	444	Predicted ORF		
ORF246	-	234122	234574	150	Hypothetical protein	A0A158CNM9	2.21E-11
ORF247	-	234635	235936	433	Virion structural protein	A0A158CNH2	3.72E-31
ORF248	+	236122	238005	627	T4-like DNA polymerase	A0A158CQK8	0
ORF249	+	238077	238574	165	Cof hydrolase	A0A0K0PVE7	1.08E-38
ORF250	-	238633	238995	120	Nuclease (SNase domain-containing protein)	A0A0H4BVY0	6.27E-19
ORF251	-	239536	239976	146	Hypothetical protein	A0A158CNR1	5.64E-09
ORF252	-	239976	241622	548	Hypothetical protein	A0A158CNU8	3.07E-92
ORF253	-	241636	242427	263	Hypothetical protein	A0A158CNW9	3.47E-11
ORF254	+	242607	243485	292	Predicted ORF		
ORF255	+	243495	244823	442	Putative RNA polymerase beta prime subunit	A0A158CNJ7	4.23E-152
ORF256	-	245213	245860	215	Hypothetical protein	A0A0S4NSA1	8.71E-92
ORF257	-	246146	246445	99	Predicted ORF		
ORF258	+	246749	246973	74	Predicted ORF		
ORF259	+	247069	247428	119	Hypothetical protein	B5ZX55	2.02E-17
ORF260	+	247539	248009	156	Hypothetical protein	R4KMJ1	7.37E-07
ORF261	+	248083	248859	258	Hypothetical protein	A0A158CNH8	3.52E-22
ORF262	+	248951	249796	281	Putative peptidoglycan binding domain protein	A0A0A8J8S6	6.06E-50
ORF263	-	249863	250798	311	Predicted ORF		
ORF264	-	250864	252417	517	RAD2/SF2 helicase	A0A158APK7	5.48E-85
ORF265	-	252459	253118	219	Hypothetical protein		
ORF266	+	253164	253979	271	Poly(3-hydroxyalkanoate) depolymerase	A0A069PPN5	2.65E-117
ORF267	-	254050	254484	144	Predicted ORF		
ORF268	-	254520	254954	144	Putative N-acetyltransferase	A0A0K2QQJ6	4.29E-42
ORF269	-	255008	255418	136	Hypothetical protein	A0A0Q4N9E9	8.27E-06
ORF270	-	255384	255689	101	Hypothetical protein	A0A158EGG2	1.28E-15
ORF271	-	255742	257784	680	Putative RNA polymerase beta prime subunit	A0A158AQG9	9.04E-177
ORF272	-	257842	260145	767	DNA-directed RNA polymerase subunit beta	A0A158APS3	0
ORF273	+	260334	260678	114	Hypothetical protein	A0A158API7	2.86E-08
ORF274	-	260769	262511	580	Hypothetical protein	A0A158ARQ9	3.40E-37
ORF275	-	262573	264276	567	Hypothetical protein	A0A0A8J8I3	4.16E-11
ORF276	-	264343	266064	573	Hypothetical protein	A0A0K2QRM1	3.20E-31
ORF277	-	266235	266951	238	Hypothetical protein	A0A158APF8	5.91E-17
ORF278	-	267020	267850	276	Hypothetical protein	A0A158AQB4	1.91E-57
ORF279	-	267854	269059	401	Nuclease SbcCD, D subunit	A0A158APM3	1.41E-102
ORF280	-	269043	269621	192	Hypothetical protein	A0A0A8JBH1	4.18E-11
ORF281	-	269703	270281	192	Predicted ORF		
ORF282	-	270274	270678	134	Hypothetical protein	A0A158APS2	5.13E-21
ORF283	-	270785	271078	97	Hypothetical protein	L2EE75	2.10E-14
ORF284	-	271145	271768	207	Hypothetical protein	A0A158AP93	2.11E-26
ORF285	-	271810	273327	505	DNA-directed RNA polymerase subunit beta	A0A158APA7	8.39E-134
ORF286	-	273445	275526	693	Hypothetical protein	A0A158APF9	4.38E-48
ORF287	+	275863	276945	360	Hypothetical protein	A0A158AP78	5.53E-69
