# Supplementary material for: Replications of Two Closely Related Groups of Jumbo Phages Show Different Level of Dependence on Host-encoded RNA Polymerase
Source: Front Microbiol. 2017 Jun 13;8:1010. doi: 10.3389/fmicb.2017.01010 (PMC5468394; doi:10.3389/fmicb.2017.01010)
Supplement: Supplementary file 3 [file Table3.PDF]

Table S2B. Annotation of phiRP31 ORFs.

ORF	strand	start	end	length (aa)	Annotation	Uniprot ID	e value
ORF1	-	30	452	140	MutT/nudix family protein	Q5QW66	1.39E-33
ORF2	-	501	2687	728	T4-like DNA polymerase	A0A158AP48	0
ORF3	-	2789	3235	148	Predicted ORF		
ORF4	-	3673	5490	605	Predicted ORF		
ORF5	-	5492	5899	135	Hypothetical protein	A0A158ARA6	2.79E-32
ORF6	-	6009	6251	80	Predicted ORF		
ORF7	-	6268	6810	180	Predicted ORF		
ORF8	-	6833	7228	131	Predicted ORF		
ORF9	-	7235	7669	144	Predicted ORF		
ORF10	-	7757	8272	171	Predicted ORF		
ORF11	-	8269	9087	272	Predicted ORF		
ORF12	-	9101	9472	123	Predicted ORF		
ORF13	-	9598	10602	334	Hypothetical protein	A0A158ANJ8	4.46E-50
ORF14	-	10691	11170	159	Predicted ORF		
ORF15	+	11556	12359	267	Hypothetical protein	A0A158ANV0	4.53E-99
ORF16	-	12411	12974	187	Predicted ORF		
ORF17	-	12977	13534	185	Predicted ORF		
ORF18	-	13612	14172	186	Predicted ORF		
ORF19	-	14485	15006	173	Predicted ORF		
ORF20	-	15735	16352	205	Predicted ORF		
ORF21	+	16980	17585	201	Predicted ORF		
ORF22	+	17714	18979	421	Hypothetical protein	A0A158ANF9	4.60E-127
ORF23	-	19035	21248	737	Terminase, large subunit	A0A158AN67	0
ORF24	-	21400	23109	569	Virion structural protein	A0A158ANU9	1.48E-115
ORF25	-	23173	25761	862	Putative virion stractural protein	A0A158ANN8	0
ORF26	-	25846	26769	307	Hypothetical protein	A0A158APH4	9.54E-52
ORF27	+	27225	29315	696	Putative tail sheath	A0A158AN48	0
ORF28	+	29391	30311	306	Putative major virion structural protein	A0A158ANS9	9.11E-88
ORF29	-	30434	31171	245	Hypothetical protein	R9MDL3	9.45E-08
ORF30	-	31174	31470	98	Predicted ORF		
ORF31	-	31706	32209	167	Predicted ORF		
ORF32	-	32297	32491	64	Predicted ORF		
ORF33	-	32557	33330	257	Predicted ORF		
ORF34	-	33561	34334	257	Putative virion stractural protein	A0A0K2QQL0	2.17E-76
ORF35	-	34341	35063	240	Hypothetical protein	A0A158ANQ2	1.05E-78
ORF36	-	35074	36552	492	Hypothetical protein	A0A158ANE4	2.85E-57
ORF37	-	36556	36729	57	Predicted ORF		
ORF38	-	36849	41297	1482	Putative RNA polymerase beta subunit	A0A158ANL1	0
ORF39	-	41300	43294	664	Putative RNA polymerase beta prime subunit	A0A158ANM3	0
ORF40	+	43412	50920	2502	Putative soluble lytic murein transglycosylase	A0A0A8J8P3	3.51E-56
ORF41	+	50999	53113	704	Hypothetical protein	A0A158ANA8	1.24E-128
ORF42	+	53149	53499	116	RyR domain protein	A0A0A0YVI6	1.68E-43
ORF43	-	53558	54787	409	Hypothetical protein	A0A158ANK2	2.20E-18
ORF44	-	54872	55420	182	Hypothetical protein	A0A158ANA0	1.21E-42
ORF45	-	55445	56290	281	Hypothetical protein	A0A158AQI4	1.47E-08
ORF46	-	56362	56838	158	Predicted ORF		
ORF47	-	56962	57369	135	Predicted ORF		
ORF48	+	57472	57990	172	Predicted ORF		
ORF49	-	57987	58958	323	Hypothetical protein	A0A0K2QR89	1.13E-21
ORF50	-	58906	59556	216	Predicted ORF		
ORF51	-	59558	60232	224	Virion structural protein	A0A158ANL7	9.71E-71
ORF52	-	60251	60538	95	Putative GTP-binding protein	I4IU58	2.37E-12
ORF53	-	60812	61585	257	Phosphate starvation-inducible protein PhoH	A0A0V7ZT44	6.39E-57
ORF54	-	61899	62786	295	Predicted ORF		
ORF55	-	62856	64262	468	Putative UvsX protein	A0A158ANI6	5.60E-174
ORF56	+	64550	65365	271	Hypothetical protein	A0A158ANA1	9.99E-10
ORF57	-	65447	65797	116	Hypothetical protein	Q8SD08	3.76E-07
ORF58	-	65936	67537	533	Ribonuclease H	A0A158AN34	5.46E-59
ORF59	-	67627	68043	138	Predicted ORF		
ORF60	-	68040	68465	141	Predicted ORF		
ORF61	+	68550	69848	432	Putative virion stractural protein	A0A158AQ83	1.43E-85
ORF62	+	69863	70495	210	Hypothetical protein	A0A158ANE1	1.85E-43
ORF63	-	70547	71485	312	Hypothetical protein	A0A158AMW8	4.55E-53
ORF64	+	71484	71603	39	Predicted ORF		
ORF65	+	71600	74092	830	Putative SbcC-ATPase	A0A158AMV9	9.51E-137
ORF66	-	74134	74688	184	Hypothetical protein	A0A0A8J8X9	3.07E-17
ORF67	-	74709	75965	418	Virion structural protein	A0A158AMX0	8.33E-79
ORF68	+	76162	77766	534	YomR	A0A158AMM8	1.68E-109
ORF69	+	77819	79096	425	YomR	W8CZP9	1.66E-94
ORF70	+	79172	79549	125	Predicted ORF		
ORF71	+	79830	80717	295	Predicted ORF		
ORF72	+	80787	82400	537	Radical SAM domain-containing protein	U2PWE8	3.22E-14
ORF73	+	82414	83496	360	Arylsulfatase regulator (Fe-S oxidoreductase)	G4Q4C0	7.56E-07
ORF74	+	83493	84641	382	Predicted ORF		
ORF75	+	84638	85897	419	Molybdenum cofactor biosynthesis protein A	A0A0P1F1H8	1.04E-29
ORF76	+	85906	86742	278	Mangotoxin biosynthesis-involved protein MgoB	A0A0X1T461	9.30E-62
ORF77	-	86793	87440	215	Predicted ORF		
ORF78	-	87605	88219	204	Hypothetical protein	A0A0A8J9A2	1.04E-21
ORF79	-	88322	88603	93	Predicted ORF		
ORF80	-	88654	88992	112	Predicted ORF		

ORF81	−	89008	89472	154	Predicted ORF		
ORF82	−	89546	90139	197	Crossover junction endodeoxyribonuclease RuvC	A0A158AMN9	6.02E−35
ORF83	−	90148	91032	294	Virion structural protein	A0A158APY6	3.92E−69
ORF84	−	91111	93357	748	Putative virion stractural protein	A0A158AM86	6.65E−140
ORF85	+	93446	96250	934	Putative virion stractural protein	A0A158AMM1	0
ORF86	+	96247	96471	74	Predicted ORF		
ORF87	−	96692	98812	706	Predicted ORF		
ORF88	−	98891	100567	558	Putative RNA polymerase beta subunit	A0A158AMP5	2.48E−135
ORF89	−	100620	101774	384	Hypothetical protein	A0A158APR4	5.27E−08
ORF90	−	102057	104216	719	Putative major head protein	A0A158AMG1	6.61E−174
ORF91	−	104266	104829	187	Hypothetical protein	A0A158AM88	1.82E−06
ORF92	+	105054	106589	511	Putative DnaB helicase	A0A158AMU7	1.58E−165
ORF93	−	106650	107441	263	Hypothetical protein	A0A0C5K996	2.58E−45
ORF94	−	107581	108156	191	Predicted ORF		
ORF95	−	108247	108594	115	Hypothetical protein	A0A0D0HCC4	3.72E−23
ORF96	−	108673	109098	141	Hypothetical protein	Q2Z0W1	1.13E−08
ORF97	−	109157	109357	66	Predicted ORF		
ORF98	+	109356	109466	36	Predicted ORF		
ORF99	−	109467	109844	125	Hypothetical protein	A0A0D0FRY7	9.60E−48
ORF100	−	109907	110704	265	Predicted ORF		
ORF101	−	110766	111764	332	Predicted ORF		
ORF102	−	111956	112882	308	Predicted ORF		
ORF103	+	113047	113334	95	Predicted ORF		
ORF104	−	113822	114250	142	Predicted ORF		
ORF105	−	114291	114626	111	Predicted ORF		
ORF106	−	114781	115131	116	Predicted ORF		
ORF107	−	115194	115454	86	Predicted ORF		
ORF108	−	115548	115874	108	Predicted ORF		
ORF109	−	115890	116030	46	Predicted ORF		
ORF110	−	116047	116379	110	Predicted ORF		
ORF111	−	116390	116767	125	Predicted ORF		
ORF112	−	116843	117274	143	Predicted ORF		
ORF113	−	117490	118293	267	Predicted ORF		
ORF114	−	118290	119288	332	Hypothetical protein		
ORF115	−	119367	120272	301	Predicted ORF		
ORF116	−	120394	120750	118	Predicted ORF		
ORF117	−	120918	121580	220	Predicted ORF		
ORF118	−	121683	122039	118	Predicted ORF		
ORF119	−	122114	122503	129	Predicted ORF		
ORF120	−	122531	123886	451	Mitochondrial chaperone BCS1	A0A0W4ZVQ0	1.25E−20
ORF121	−	123984	124592	202	Predicted ORF		
ORF122	−	124613	125248	211	Hypothetical protein	W6ARW1	3.96E−27
ORF123	−	125361	125711	116	Hypothetical protein	Q7NXS4	5.22E−36
ORF124	−	125759	126394	211	Predicted ORF		
ORF125	−	127312	127437	41	Predicted ORF		
ORF126	−	127434	127625	63	Predicted ORF		
ORF127	−	127622	127852	76	Hypothetical protein	A0A158E9B3	1.04E−10
ORF128	−	127849	128301	150	Hypothetical protein	A0A106QCE2	2.08E−46
ORF129	−	129289	130164	291	Predicted ORF		
ORF130	−	130305	130655	116	Predicted ORF		
ORF131	−	130747	131349	200	Deoxycytidine triphosphate deaminase	A0A0Q5FHS0	6.79E−58
ORF132	−	131393	131527	44	Predicted ORF		
ORF133	−	131795	133597	600	Hypothetical protein	F8SJ78	1.23E−86
ORF134	−	133608	133964	118	Predicted ORF		
ORF135	−	134083	135123	346	Hypothetical protein	H8ZN24	8.52E−42
ORF136	−	135116	136156	346	Hypothetical protein	E3SQ11	1.66E−71
ORF137	−	136157	136768	203	Hypothetical protein	M4PM32	1.18E−09
ORF138	−	136819	137295	158	Predicted ORF		
ORF139	−	137296	138174	292	Predicted ORF		
ORF140	−	138234	138746	170	Hypothetical protein	A0A081BFQ3	9.02E−09
ORF141	−	138895	139503	202	Predicted ORF		
ORF142	−	139820	140062	80	Predicted ORF		
ORF143	−	140179	140361	60	Predicted ORF		
ORF144	−	140674	140997	107	Predicted ORF		
ORF145	−	140998	142683	561	Hypothetical protein	M1LRP3	1.72E−06
ORF146	−	143005	144702	565	Predicted ORF		
ORF147	−	144765	145181	138	Hypothetical protein	C6XE84	5.72E−09
ORF148	−	145236	145457	73	Predicted ORF		
ORF149	−	145514	147172	552	Predicted ORF		
ORF150	−	147172	150441	1089	Putative tail fiber protein 1	M1F173	4.60E−170
ORF151	−	150455	151003	182	Hypothetical protein	A0A0A8J8X9	5.38E−07
ORF152	−	151159	152361	400	Ribonucleotide reductase of class Ia (Aerobic), beta subunit	W8X2K7	2.51E−116
ORF153	−	152499	155159	886	Ribonucleoside−diphosphate reductase	I7CD23	0
ORF154	−	155449	155904	151	Predicted ORF		
ORF155	−	155901	156482	193	Predicted ORF		
ORF156	−	156482	157132	216	Predicted ORF		
ORF157	−	157141	157587	148	Predicted ORF		
ORF158	−	157760	159721	653	DNA ligase	A0A0J1JV68	8.78E−119
ORF159	−	159718	160425	235	Predicted ORF		
ORF160	−	160518	160781	87	Predicted ORF		
ORF161	−	160846	161364	172	Predicted ORF		
ORF162	−	161434	163263	609	Hypothetical protein	K4ZBQ3	1.60E−07

ORF163	−	163433	164092	219	Transglycosylase	A0A125PA11	6.81E−21
ORF164	−	164253	164636	127	Hypothetical protein	C1A414	1.08E−35
ORF165	−	164846	165856	336	Phosphoesterase	U9U3G0	9.91E−27
ORF166	−	165946	168201	751	DEAD−like helicase	W6AS32	1.07E−157
ORF167	−	168330	168869	179	Hypothetical protein	K7HKD1	1.99E−06
ORF168	−	168887	169321	144	Predicted ORF		
ORF169	−	169358	169738	126	Hypothetical protein	A0A0Q4NJE4	2.87E−38
ORF170	−	169746	170096	116	Predicted ORF		
ORF171	−	170093	170329	78	Predicted ORF		
ORF172	−	170333	170653	106	Predicted ORF		
ORF173	+	170837	171280	147	Enoyl−CoA hydratase	A0A0Q7FG53	4.79E−36
ORF174	−	171345	171785	146	Predicted ORF		
ORF175	−	171862	172338	158	Predicted ORF		
ORF176	−	172382	172918	178	Predicted ORF		
ORF177	−	173071	173586	171	Predicted ORF		
ORF178	−	173586	174479	297	Predicted ORF		
ORF179	−	174573	174917	114	Predicted ORF		
ORF180	−	175059	175940	293	Hypothetical protein	A0A0D7KF63	2.51E−07
ORF181	−	176039	176362	107	Predicted ORF		
ORF182	−	176445	176969	174	Dihydrofolate reductase	A0A059X418	1.20E−33
ORF183	−	177012	177641	209	Hypothetical protein	C4MZR9	6.64E−08
ORF184	−	177641	178348	235	Predicted ORF		
ORF185	−	178408	179712	434	Predicted ORF		
ORF186	−	179845	180927	360	Predicted ORF		
ORF187	−	180993	181526	177	Predicted ORF		
ORF188	−	181647	182384	245	ABC−type transporter, integral membrane subunit	F2LVG9	6.73E−18
ORF189	−	182384	183172	262	ABC transporter substrate−binding protein	A0A0U3E8W3	4.59E−14
ORF190	−	183244	184164	306	TRAP transporter solute receptor like protein	B2ZXX0	3.79E−73
ORF191	−	184199	184342	47	Predicted ORF		
ORF192	−	184339	185130	263	Hypothetical protein	A0A0X3AKY0	1.23E−24
ORF193	−	185248	185730	160	Predicted ORF		
ORF194	−	185742	186098	118	Predicted ORF		
ORF195	−	186110	186460	116	Hypothetical protein	F5JC11	4.23E−31
ORF196	−	186453	186698	81	Predicted ORF		
ORF197	+	186763	187017	84	Hypothetical protein	A0A0A8J8D1	4.78E−25
ORF198	−	187057	187398	113	Predicted ORF		
ORF199	−	187519	187803	94	Predicted ORF		
ORF200	+	187907	189634	575	Hypothetical protein	G0MXP9	5.16E−08
ORF201	−	189703	190392	229	Predicted ORF		
ORF202	−	190403	191173	256	Predicted ORF		
ORF203	−	191240	192607	455	ATPase associated with various cellular activities family protein	I7DKJ3	5.82E−22
ORF204	−	192753	193178	141	Predicted ORF		
ORF205	−	193175	193501	108	Predicted ORF		
ORF206	−	193505	194797	430	Putative RNA ligase	F8SJC5	8.55E−57
ORF207	−	194803	195933	376	Hypothetical protein	K4K2V0	5.76E−17
ORF208	−	195984	197750	588	Nicotinate phosphoribosyltransferase	A0A098UDR3	9.45E−153
ORF209	−	197789	198703	304	Ribose−phosphate pyrophosphokinase	K9Z5A2	2.49E−60
ORF210	−	198709	199977	422	Putative RtcB−like protein	W6AR47	0
ORF211	−	200060	200749	229	Predicted ORF		
ORF212	−	200846	201748	300	Thymidylate synthase	S7HVU0	1.35E−116
ORF213	−	201839	202189	116	XRE family plasmid maintenance system antidote protein	A0A158E9F6	1.33E−06
ORF214	−	202192	202644	150	Predicted ORF		
ORF215	−	202655	203035	126	Predicted ORF		
ORF216	−	203039	203305	88	Predicted ORF		
ORF217	−	203380	204048	222	Predicted ORF		
ORF218	−	204233	204862	209	Thymidylate kinase	F1ZRR9	1.67E−25
ORF219	−	204959	205225	88	Predicted ORF		
ORF220	−	205227	205448	73	Predicted ORF		
ORF221	−	205530	205877	115	Predicted ORF		
ORF222	−	205907	206314	135	Hypothetical protein	A0A158CML1	1.90E−09
ORF223	−	206503	206664	53	Predicted ORF		
ORF224	−	206661	206843	60	Predicted ORF		
ORF225	−	206846	207067	73	Predicted ORF		
ORF226	−	207410	207793	127	Predicted ORF		
ORF227	−	207960	208268	102	Predicted ORF		
ORF228	−	208410	209771	453	Virion structural protein	A0A158CN86	3.86E−89
ORF229	−	209772	210449	225	Hypothetical protein	A0A158CN53	3.54E−27
ORF230	−	210500	211861	453	Putative virion stractural protein	A0A158CN49	1.65E−113
ORF231	−	211871	213454	527	Hypothetical protein	A0A158CMZ4	4.76E−71
ORF232	−	213590	215146	518	Predicted ORF		
ORF233	−	215148	216320	390	Predicted ORF		
ORF234	−	216434	217735	433	Predicted ORF		
ORF235	−	217745	219184	479	Predicted ORF		
ORF236	−	219357	220781	474	Predicted ORF		
ORF237	−	220873	222177	434	Predicted ORF		
ORF238	−	222277	224052	591	Predicted ORF		
ORF239	−	224196	224795	199	Virion structural protein	A0A158CPM5	1.02E−23
ORF240	−	224799	225695	298	Putative virion structural protein	A0A158CNH4	8.81E−32
ORF241	−	225777	227045	422	Virion structural protein	A0A158CNF7	9.41E−72
ORF242	+	227103	228260	385	Virion structural protein	A0A158CNJ1	1.92E−99
ORF243	+	228272	231199	975	Putative virion stractural protein	A0A158CNF2	0
ORF244	−	231258	232694	478	Predicted ORF		

ORF245	−	232777	234111	444	Predicted ORF		
ORF246	−	234122	234574	150	Hypothetical protein	A0A158CNM9	2.21E−11
ORF247	−	234635	235936	433	Virion structural protein	A0A158CNH2	3.72E−31
ORF248	+	236122	238005	627	T4-like DNA polymerase	A0A158CQK8	0
ORF249	+	238077	238574	165	Cof hydrolase	A0A0K0PVE7	1.08E−38
ORF250	−	238633	238995	120	Nuclease (SNase domain-containing protein)	A0A0H4BVY0	6.27E−19
ORF251	−	239536	239976	146	Hypothetical protein	A0A158CNR1	5.64E−09
ORF252	−	239976	241622	548	Hypothetical protein	A0A158CNU8	3.07E−92
ORF253	−	241636	242427	263	Hypothetical protein	A0A158CNW9	3.47E−11
ORF254	+	242607	243485	292	Predicted ORF		
ORF255	+	243495	244823	442	Putative RNA polymerase beta prime subunit	A0A158CNJ7	4.23E−152
ORF256	−	245213	245860	215	Hypothetical protein	A0A0S4NSA1	8.71E−92
ORF257	−	246146	246445	99	Predicted ORF		
ORF258	+	246749	246973	74	Predicted ORF		
ORF259	+	247069	247428	119	Hypothetical protein	B5ZX55	2.02E−17
ORF260	+	247539	248009	156	Hypothetical protein	R4KMJ1	7.37E−07
ORF261	+	248083	248859	258	Hypothetical protein	A0A158CNH8	3.52E−22
ORF262	+	248951	249796	281	Putative peptidoglycan binding domain protein	A0A0A8J8S6	6.06E−50
ORF263	−	249863	250798	311	Predicted ORF		
ORF264	−	250864	252417	517	RAD2/SF2 helicase	A0A158APK7	5.48E−85
ORF265	−	252459	253118	219	Hypothetical protein		
ORF266	+	253164	253979	271	Poly(3-hydroxyalkanoate) depolymerase	A0A069PPN5	2.65E−117
ORF267	−	254050	254484	144	Predicted ORF		
ORF268	−	254520	254954	144	Putative N-acetyltransferase	A0A0K2QQJ6	4.29E−42
ORF269	−	255008	255418	136	Hypothetical protein	A0A0Q4N9E9	8.27E−06
ORF270	−	255384	255689	101	Hypothetical protein	A0A158EGG2	1.28E−15
ORF271	−	255742	257784	680	Putative RNA polymerase beta prime subunit	A0A158AQG9	9.04E−177
ORF272	−	257842	260145	767	DNA-directed RNA polymerase subunit beta	A0A158APS3	0
ORF273	+	260334	260678	114	Hypothetical protein	A0A158API7	2.86E−08
ORF274	−	260769	262511	580	Hypothetical protein	A0A158ARQ9	3.40E−37
ORF275	−	262573	264276	567	Hypothetical protein	A0A0A8J8I3	4.16E−11
ORF276	−	264343	266064	573	Hypothetical protein	A0A0K2QRM1	3.20E−31
ORF277	−	266235	266951	238	Hypothetical protein	A0A158APF8	5.91E−17
ORF278	−	267020	267850	276	Hypothetical protein	A0A158AQB4	1.91E−57
ORF279	−	267854	269059	401	Nuclease SbcCD, D subunit	A0A158APM3	1.41E−102
ORF280	−	269043	269621	192	Hypothetical protein	A0A0A8JBH1	4.18E−11
ORF281	−	269703	270281	192	Predicted ORF		
ORF282	−	270274	270678	134	Hypothetical protein	A0A158APS2	5.13E−21
ORF283	−	270785	271078	97	Hypothetical protein	L2EE75	2.10E−14
ORF284	−	271145	271768	207	Hypothetical protein	A0A158AP93	2.11E−26
ORF285	−	271810	273327	505	DNA-directed RNA polymerase subunit beta	A0A158APA7	8.39E−134
ORF286	−	273445	275526	693	Hypothetical protein	A0A158APF9	4.38E−48
ORF287	+	275863	276945	360	Hypothetical protein	A0A158AP78	5.53E−69
