# Supplementary material for: Replications of Two Closely Related Groups of Jumbo Phages Show Different Level of Dependence on Host-encoded RNA Polymerase
Source: Front Microbiol. 2017 Jun 13;8:1010. doi: 10.3389/fmicb.2017.01010 (PMC5468394; doi:10.3389/fmicb.2017.01010)
Supplement: Supplementary file 4 [file Table4.PDF]

Table S3. Mass spectrometry data for phiRP31 virion proteins.

RP31 protein	Predicted function	Theo. mol. mas (kDa)	Obs. mol. mass (kDa)	No. of unique peptides	Sequence coverage (%)
ORF40	Putative soluble lytic murein transglycosylase	268.6	260	9	4.28
ORF38	Putative RNA polymerase beta subunit	167	180	8	6.34
ORF150	Putative tail fiber protein	111.2	120	5	5.51
ORF243	Putative virion structural protein	108.3	110	6	6.05
ORF85	Putative virion structural protein	102.7	100	14	16.17
ORF84	Putative virion structural protein	85.3	80	4	8.16
ORF166	DEAD-like helicase	85.9	80	14	17.98
ORF27	Putative tail sheath protein	76.8	75	24	32.33
ORF90	Putative major head protein	76.8	75	17	24.9
ORF286	Unknown	73.9	69	4	8.95
ORF39	Putative RNA polymerase beta prime subunit	75.2	74	26	40.96
ORF24	Virion structural protein	63.9	62	22	54.66
ORF133	Unknown	63.6	62	2	4.17
ORF36	Unknown	54.7	55	28	58.94
ORF230	Putative virion structural protein	51.9	54	10	19.65
ORF237	Unknown	46.4	46	9	16.82
ORF245	Unknown	48.6	49	2	4.28
ORF255	Putative RNA polymerase beta prime subunit	48.9	49	7	16.06
ORF242	Virion structural protein	43.6	42	2	13.51
ORF234	Unknown	46.6	38	11	29.56
ORF26	Hypothetical protein	34.3	36	9	25.73
ORF240	Putative virion structural protein	33.1	34	7	21.14
ORF28	Putative major virion structural protein	33.7	35	4	16.01
ORF56	Unknown	29.9	28	6	22.88
ORF192	Unknown	29.1	27	1	3.8
ORF51	Virion structural protein	24.6	25	4	12.05
ORF124	Unknown	24.5	25	2	8.53
ORF78	Unknown	22.9	23	6	4.96
ORF62	Unknown	22.8	23	3	5.15
ORF239	Virion structural protein	22.9	23	4	7.33
ORF140	Unknown	17.1	18	2	10.59

ORF213	XRE family plasmid maintenance system antidote protein	12.8	14	1	9.48
