# Supplementary material for: Replications of Two Closely Related Groups of Jumbo Phages Show Different Level of Dependence on Host-encoded RNA Polymerase
Source: Front Microbiol. 2017 Jun 13;8:1010. doi: 10.3389/fmicb.2017.01010 (PMC5468394; doi:10.3389/fmicb.2017.01010)
Supplement: Supplementary file 5 [file Presentation1.pptx]

## Slide 1
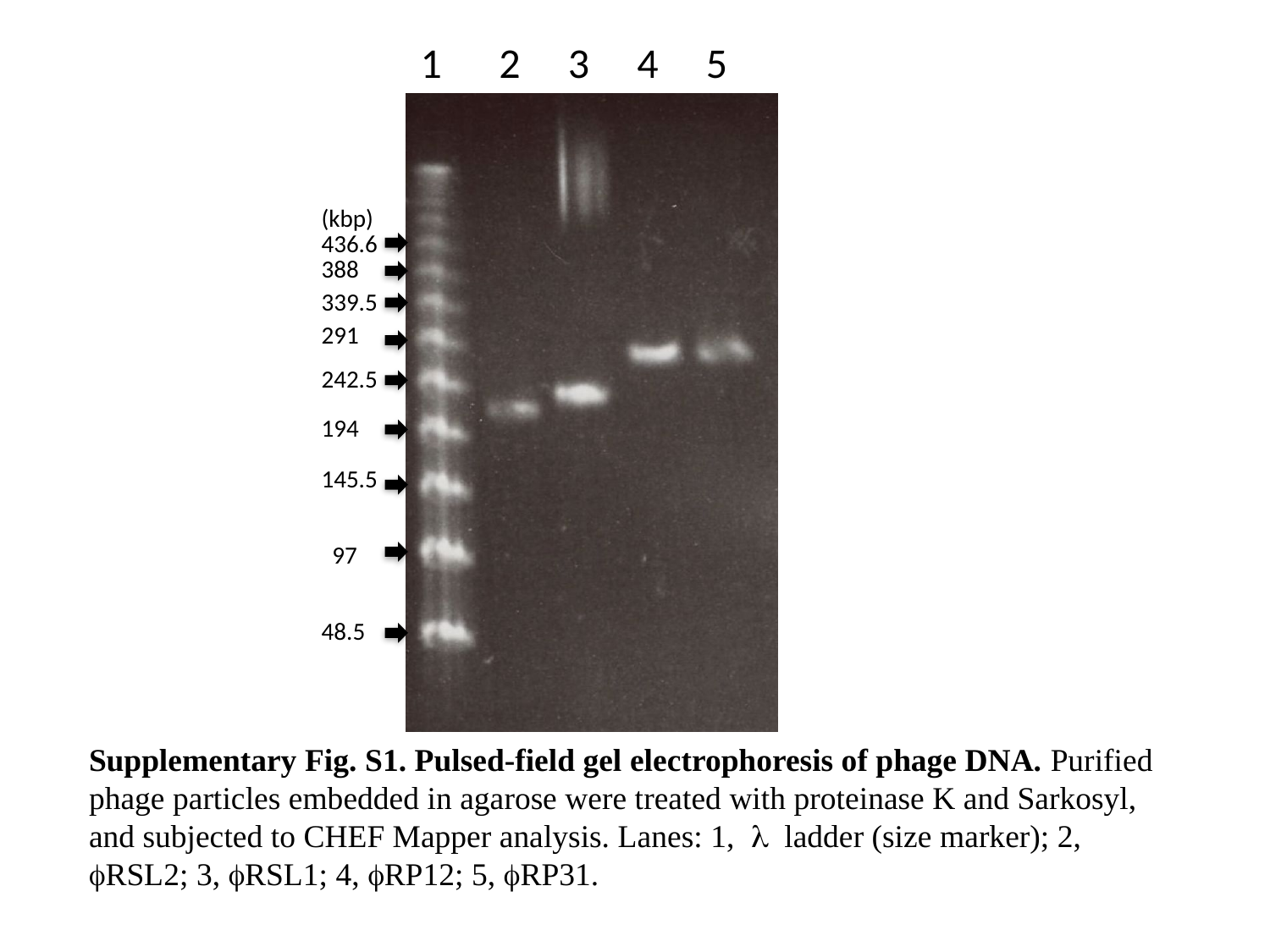

1 2 3 4 5
(kbp)
436.6
388
339.5
291
242.5
194
145.5
 97
48.5
Supplementary Fig. S1. Pulsed-field gel electrophoresis of phage DNA. Purified phage particles embedded in agarose were treated with proteinase K and Sarkosyl, and subjected to CHEF Mapper analysis. Lanes: 1, l ladder (size marker); 2, fRSL2; 3, fRSL1; 4, fRP12; 5, fRP31.

## Slide 2
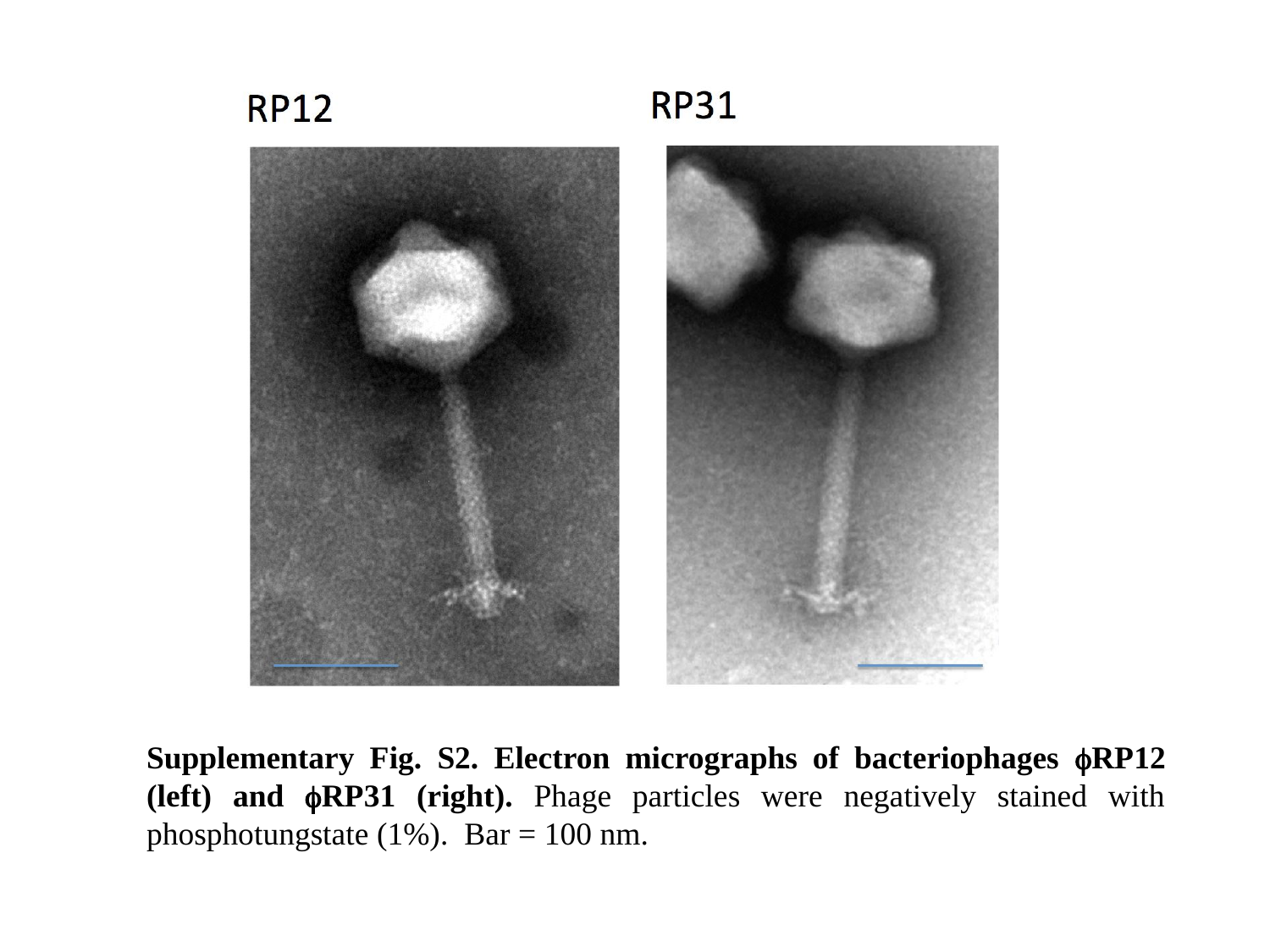

Supplementary Fig. S2. Electron micrographs of bacteriophages fRP12 (left) and fRP31 (right). Phage particles were negatively stained with phosphotungstate (1%). Bar = 100 nm.

## Slide 3
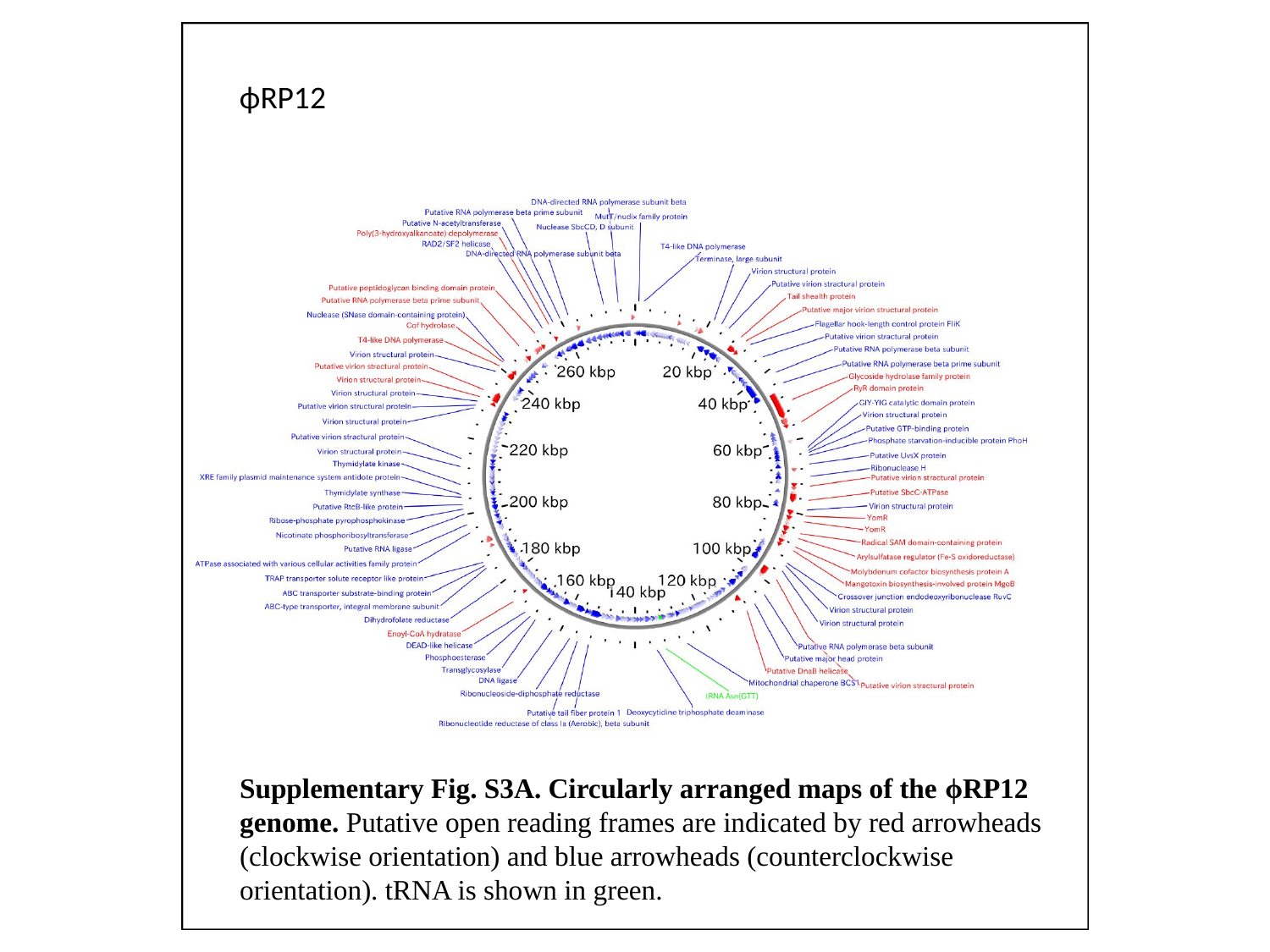

ϕRP12
Supplementary Fig. S3A. Circularly arranged maps of the ϕRP12 genome. Putative open reading frames are indicated by red arrowheads (clockwise orientation) and blue arrowheads (counterclockwise orientation). tRNA is shown in green.

## Slide 4
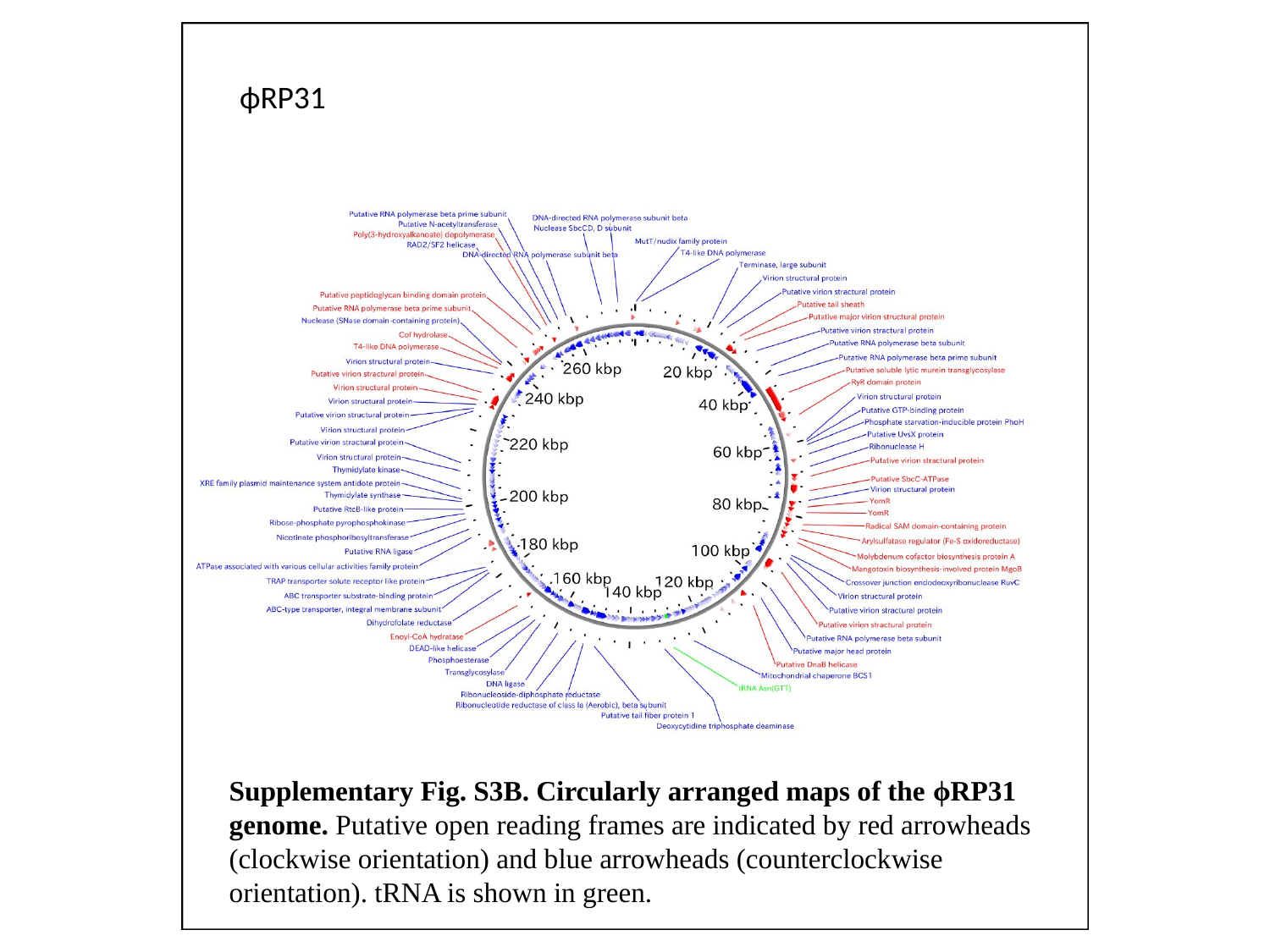

ϕRP31
Supplementary Fig. S3B. Circularly arranged maps of the ϕRP31 genome. Putative open reading frames are indicated by red arrowheads (clockwise orientation) and blue arrowheads (counterclockwise orientation). tRNA is shown in green.

## Slide 5
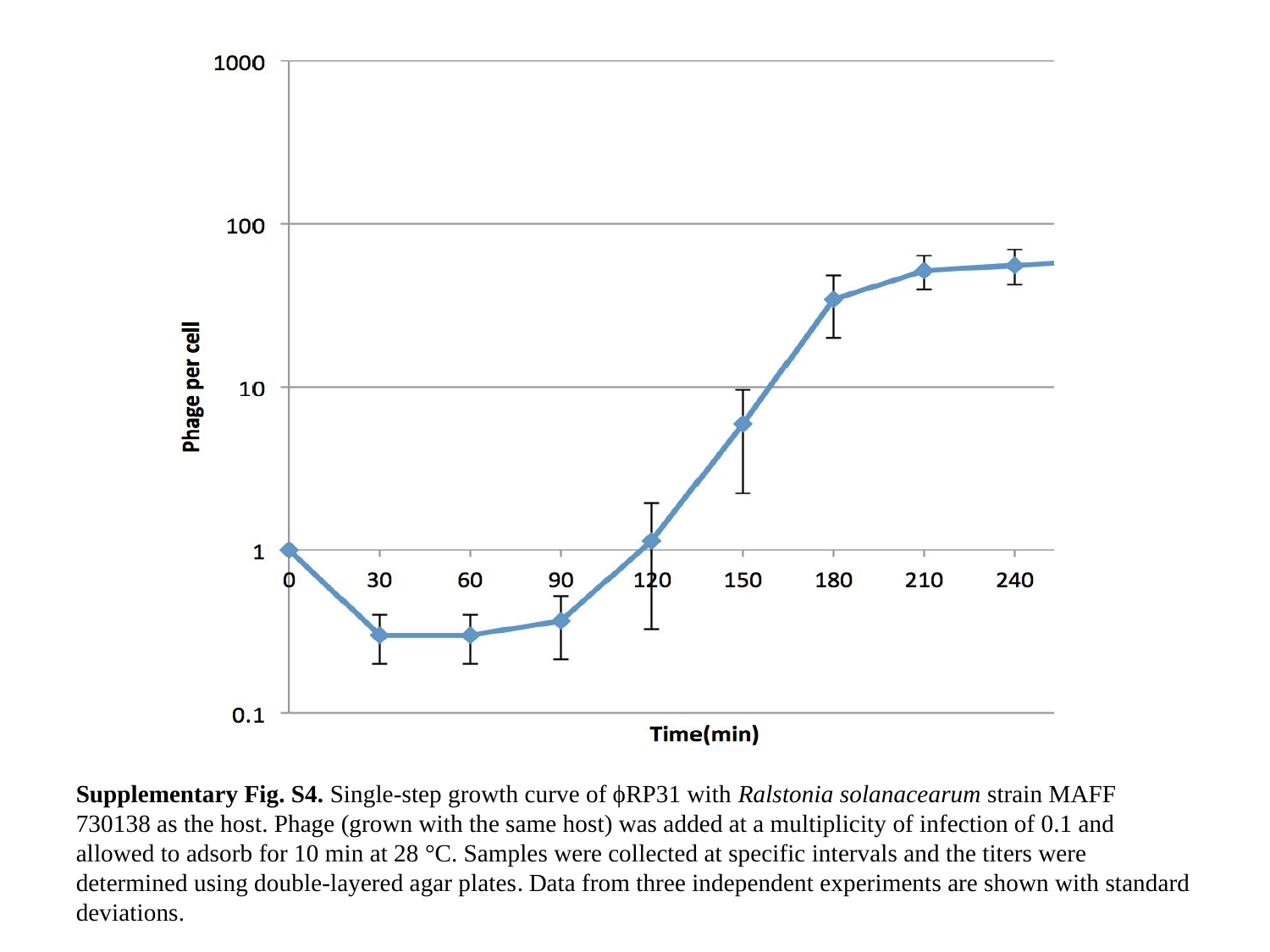

Supplementary Fig. S4. Single-step growth curve of ϕRP31 with Ralstonia solanacearum strain MAFF 730138 as the host. Phage (grown with the same host) was added at a multiplicity of infection of 0.1 and allowed to adsorb for 10 min at 28 °C. Samples were collected at specific intervals and the titers were determined using double-layered agar plates. Data from three independent experiments are shown with standard deviations.
